# Prediction of tissue exposures of polymyxin-B, amikacin and sulbactam using physiologically-based pharmacokinetic modeling

**DOI:** 10.3389/fmicb.2024.1435906

**Published:** 2024-10-07

**Authors:** Mengyuan Wu, Kun Feng, Xiao Wu, Chang Liu, Shixing Zhu, Frederico S. Martins, Mingming Yu, Zhihua Lv, Meixing Yan, Sherwin K. B. Sy

**Affiliations:** ^1^School of Medicine and Pharmacy, Ocean University of China, Qingdao, China; ^2^Women and Children Hospital, Qingdao University, Qingdao, China; ^3^Qingdao Central Hospital, University of Health and Rehabilitation Sciences, Qingdao, China; ^4^Department of Clinical and Toxicological Analysis, Faculty of Pharmaceutical Sciences, Universidade de São Paulo, São Paulo, Brazil; ^5^Department of Statistics, Universidade Estadual de Maringá, Maringá, Paraná, Brazil

**Keywords:** antibiotic combination, tissue exposure, physiologically-based pharmacokinetic, pharmacodynamic index, multidrug resistance (MDR)

## Abstract

**Background:**

The combination antimicrobial therapy consisting of amikacin, polymyxin-B, and sulbactam demonstrated *in vitro* synergy against multi-drug resistant *Acinetobacter baumannii*.

**Objectives:**

The objectives were to predict drug disposition and extrapolate their efficacy in the blood, lung, heart, muscle and skin tissues using a physiologically-based pharmacokinetic (PBPK) modeling approach and to evaluate achievement of target pharmacodynamic (PD) indices against *A. baumannii*.

**Methods:**

A PBPK model was initially developed for amikacin, polymyxin-B, and sulbactam in adult subjects, and then scaled to pediatrics, accounting for both renal and non-renal clearances. The simulated plasma and tissue drug exposures were compared to the observed data from humans and rats. Efficacy was inferred using joint probability of target attainment of target PD indices.

**Results:**

The simulated plasma drug exposures in adults and pediatrics were within the 0.5 to 2 boundary of the mean fold error for the ratio between simulated and observed means. Simulated drug exposures in blood, skin, lung, and heart were consistent with reported penetration ratio between tissue and plasma drug exposure. In a virtual pediatric population from 2 to <18 years of age using pediatric dosing regimens, the interpretive breakpoints were achieved in 85–90% of the population.

**Conclusion:**

The utility of PBPK to predict and simulate the amount of antibacterial drug exposure in tissue is a practical approach to overcome the difficulty of obtaining tissue drug concentrations in pediatric population. As combination therapy, amikacin/polymyxin-B/sulbactam drug concentrations in the tissues exhibited sufficient penetration to combat extremely drug resistant *A. baumannii* clinical isolates.

## 1 Introduction

The overuse of antibiotics has led to the emergence of multidrug-resistant (MDR) *Acinetobacter baumannii*, which made treatment of related infections increasingly difficult; consequently, antibiotic monotherapies are no longer effective in the clinic (Oo and Sy, 2020; Oo et al., 2023). With increasing incidences of MDR *A. baumannii*, polymyxins became the last-line antibiotic agent (Li and Nation, 2006; Li et al., 2006). Polymyxin-B combination with other antibiotics exhibited better efficacy against MDR *A. baumannii* than individual antibiotic monotherapies (Rao et al., 2016; Liu et al., 2023). Our laboratory previously evaluated the *in vitro* synergistic activities of polymyxin-B, amikacin and sulbactam combination therapy against 11 MDR *A. baumannii* and showed that this triple combination restricted the mutant selection window and reduced the opportunity for the bacteria to develop further resistance (Zhu et al., 2022a). Using metabolomic profiling, we further demonstrated that this triple antibiotic combination significantly disrupted cellular outer membrane structures, fatty acids, glycerophospholipids, nucleotide and peptide metabolic pathways in MDR *A. baumannii* (Zhu et al., 2023). Sulbactam and polymyxin-B disrupted the integrity and stability of the cell wall and outer membrane by affecting peptidoglycans and lipopolysaccharides (Zhang and Rock, 2008), allowing companion antibiotics such as amikacin to enter the bacteria cell and inhibit protein synthesis that leads to cell death (Taber et al., 1987; Kotra et al., 2000).

The interpretive criteria for antimicrobial susceptibility are based on drug exposures in the blood, as it is difficult to obtain drug exposure data in the tissues. *A. baumannii* infections in critically ill patients in intensive care unit are often due to pneumonia, endocarditis, skin and bloodstream infections (Sopirala et al., 2008; Lagana et al., 2015; Abdi et al., 2020); these infections often occur in tissues where drug concentrations are often lower than that in the blood. Physiologically-based pharmacokinetic (PBPK) models provide a non-invasive alternative to extrapolate drug efficacy in the infected tissues (Martins et al., 2020; Martins et al., 2021; Zhu et al., 2022b; Martins et al., 2023; Zhang et al., 2023) and provides the necessary tool to evaluate sufficiency of exposure in the target organs.

Even though the pharmacokinetics of polymyxin-B, amikacin, and sulbactam have been reported in pediatrics (Kafetzis et al., 1979; Schaad et al., 1986; Xu et al., 2022), drug exposure data were lacking in the above tissues. In this study, we developed robust PBPK models and linked them to an exposure-based pharmacodynamic (PD) assessment to explore the adequacy of drug exposure in these organs. The present study aimed to predict drug exposure in the blood, lung, skin, and heart using clinical dosing regimens of polymyxin-B, amikacin and sulbactam for combination therapy.

## 2 Materials and methods

### 2.1 Clinical data used for PBPK model development and evaluation

The model of sulbactam was developed and evaluated in our previous study (Zhu et al., 2022b); it was not thoroughly evaluated in the current study. The clinical data including pharmacokinetic profiles and exposure parameters of polymyxin-B and amikacin were obtained from search results in Web of Science and PubMed databases. The demographic information in the literature was summarized by gender, age, weight, renal function, dosing regimen and drug exposure. Chart extraction data tool, Web Plot Digitizer (version 4.5 https://automeris.io/wpd) was used to extract drug exposure data from the literature. Those extracted data were from critically ill patients and healthy volunteers, who received either single or multiple dosing regimens via intravenous bolus or infusion and were used to optimize key parameters of the PBPK model. Adult PBPK model was developed, qualified and then scaled to the pediatric population. The extracted data of pediatrics from the literature were used to validate the pediatric PBPK model.

### 2.2 The development of adult PBPK model

The PBPK software, PK-Sim^®^ (Version 10.0; part of the Open Systems Pharmacology Suite, https://www.open-systems-pharmacology.org), was used to develop the adult model of polymyxin-B and amikacin. The standard distribution model assumes four sub-compartments per organ, which included compartments for blood cells, plasma, interstitial space, and intracellular space (Bischoff, 1986). Interstitial fluid in tissues is the medium for infection transmission, and also the medium for antibiotics to be distributed at the infection site (Segal et al., 1990); the tissue drug concentrations we assessed were taken from the interstitial fluid compartment. We used the parameter identification module to optimize and then select the partition coefficient method.

The physicochemical characteristics and physiological parameters of polymyxin-B and amikacin applied in model development were obtained from drug bank^1^ and the literature as listed in Table 1. Demographical information was applied in the model development including age, gender, and weight, in addition to dosing regimens and plasma concentration-time profiles. In order to ensure consistency of the simulated drug exposure distribution in the tissues with those reported in the literature, we adjusted the standard deviation of the partition coefficients by comparing the inter-individual variability of drug tissue exposure with that reported in the literature (Zhu et al., 2022b).

**Table 1:** Drug characteristics and parameters of polymyxin-B/amikacin used in PBPK model building.

Parameter	Amikacin	Polymyxin-B
Physicochemical characteristics		
Molecular weight (g/mol)	585.6	1203.5
Compound type	Ampholyte	Ampholyte
Solubility (mg/mL)^a^	49.7	0.0744
pKa acid^a^	12.1	11.6
pKa base^a^	9.79	10.2
Lipophilicity (logP)^a^	−3.2	−0.89
Distribution (WB-PBPK)		
Partition coefficients^c^	Schmitt: heart:1.10 ± 0.09; lung: 0.50 ± 0.04; skin:0.41 ± 0.12	PK-Sim standard: heart:1.03 ± 0.31; lung: 2.83 ± 1.10; skin:1.53 ± 0.45
ƒ_u_ (adults)	0.90^a^	0.80^a^
ƒ_u_ (pediatrics)	0.72	0.50
B:P ratio ^c^	0.82	0.56
Protein binding partner	Albumin (Gamba et al., 1990)	α_1_-acid glycoprotein (Zavascki et al., 2008)
Elimination		
CL_renal_ (mL/min/kg)	1.4^b^ (Vogelstein et al., 1977; Bauer et al., 1980; Lanao et al., 1981; Garraffo et al., 1990; Vanhaeverbeek et al., 1993; Mahmoudi et al., 2013)	0.002^b^ (Zavascki et al., 2007; Zavascki et al., 2008; Manchandani et al., 2016)
CL_non-renal_ (mL/min/kg)	–	0.67^b^ (Zavascki et al., 2008; Sandri et al., 2013a; Sandri et al., 2013b; Burkin et al., 2021; Xu et al., 2022)
CL_biliary_ (mL/min/kg)	–	0.001^b^ (Manchandani et al., 2016)
GFR I (mL/min)	≥60	≥60
GFR II (mL/min)	40–59	40–59
GFR II (mL/min)	30–39	30–39

WB-PBPK, whole-body physiologically based pharmacokinetic; CL, clearance; f_u_, fraction unbound; GFR, glomerular filtration rate.

a Values from www.drugbank.ca.

b Optimized based on the reported information.

c Parameter determined by PK-Sim.

The clearance of polymyxin-B was minimally affected by renal function. Consequently, the dosing regimens of polymyxin-B were not adjusted based on renal function. Less than 1% unchanged polymyxin-B was recovered in human urine; due to tubular reabsorption, the net renal clearance was in the range of 0.00032–0.0039 mL/min/kg in humans (Zavascki et al., 2007; Zavascki et al., 2008; Manchandani et al., 2016). The specific mechanism for its metabolism is still not fully understood (Avedissian et al., 2019); the non-renal clearance parameter of the polymyxin-B model was set as its total clearance and then optimized in PK-Sim (Zavascki et al., 2008; Sandri et al., 2013a; Sandri et al., 2013b; Burkin et al., 2021; Xu et al., 2022). Biliary excretion could be one of the elimination routes, but the value was not provided in the literature (Manchandani et al., 2016); biliary elimination was optimized by the parameter identification module in PK-Sim.

Amikacin is the second most commonly used antibiotic in neonatal intensive care units (Spitzer et al., 2010). Like other aminoglycosides, amikacin is primarily eliminated by glomerular filtration; a high recovery rate of its unchanged form was detected in the urine (Lanao et al., 1981). The model assumed no hepatic metabolism and renal clearance is the primary route of elimination. The reported clearance of amikacin was used in the model (Vogelstein et al., 1977; Bauer et al., 1980; Lanao et al., 1981; Garraffo et al., 1990; Vanhaeverbeek et al., 1993; Mahmoudi et al., 2013). The physiological parameters of amikacin are listed in Table 1.

To account for changes in amikacin clearance in human population with renal insufficiency, the renal physiological parameters including renal blood flow, kidney volume, hematocrit, small intestinal transit and renal perfusion were adjusted accordingly (Malik et al., 2020).

The potential risk of drug–drug interactions was considered in combination antibiotic therapy. Amikacin and sulbactam have a high renal clearance rate, while polymyxin B is primarily reabsorbed in the renal tubular cells and cleared through non-renal pathways. These drugs are not metabolized by the liver and drug–drug interaction affecting their pharmacokinetics is unlikely. However, nephrotoxicity is a concern for the combination therapy. Wang et al. reported that the amikacin/polymyxin B combination did not lead to acute kidney injury during a 30-day treatment period (Wang et al., 2021). Furthermore, *in vitro* susceptibility results indicated that combining these three antibiotics significantly reduced the drug concentration required to combat multidrug-resistant *A. baumannii*, while monotherapy would likely result in therapeutic failure.

### 2.3 Adult PBPK model evaluation

The performance of the simulations for the two antibiotics was assessed via the mean fold error (MFE, Equation 1) (Biesdorf et al., 2019):


(1) $$MFE=\frac{PK\;\mathrm{paramete}r_{\mathrm{predicted mean}}}{PK\;\mathrm{paramete}r_{\mathrm{observed mean}}}$$

MFE was performed by comparing simulated to observed maximum drug concentration (*C*_max_) and the area under the concentration-time curve (AUC). The PBPK models for adults and pediatrics were accepted when the predicted to observed PK data were within 2-fold range (i.e., 0.5 ≤ MFE ≤ 2.0).

The observed *C*_max_ and AUC were extracted from pharmacokinetic profiles reported in the literature. A virtual population containing 100 subjects with a 50:50 male-to-female ratio was established for the three antibiotics to simulate their blood and tissue drug concentrations.

### 2.4 Pediatric PBPK model development and evaluation

#### 2.4.1 Physiological parameters in the pediatric population

In general, renal function is considered fully developed by 2 years of age. For polymyxin-B, the renal function does not affect its clearance whereas the clearances of amikacin and sulbactam are influenced by renal function. Given that the incidence of renal function impairment in pediatrics is low, a virtual pediatric population from 0 to 17 years was established assuming normal renal function. Both renal and non-renal eliminations were scaled by age-dependent maturation of organ weight.

Anatomic and physiological parameters for pediatrics such as organ volumes, and composition, blood flows, protein binding and maturation of elimination processes were used in the pediatric virtual population development. These parameters were summarized from previous studies and incorporated into PK-Sim’s ontogeny database (Poulin et al., 2001; Valentin, 2002; Ince et al., 2019). Weight-based dosing is used for pediatrics. The inter-individual variability of body weight by age and gender of pediatrics was compared to an age-matched polynomial function of body weight distribution in the literature (Sy et al., 2014). In addition, a method for protein binding prediction in small children was used to predict the unbound fraction of the antibiotics (McNamara and Alcorn, 2002).

Ince et al. (2021) established and validated a PBPK model for 10 small molecule compounds in adults, including amikacin and successfully extrapolated it to pediatric populations; however, they did not investigate drug exposure in the tissues. Claassen et al. (2015) incorporated factors such as renal and hepatic maturation (including individual hepatic enzyme development) into a PBPK model for premature infants, evaluating the model’s performance for amikacin and paracetamol. They compared the predicted plasma concentration-time profiles of the two drugs with observed *in vivo* data in the blood and simulated concentrations across a wide range of gestational and postnatal ages, providing reference information for clinical use in premature pediatric populations (Claassen et al., 2015). Darlow et al. (2024) established a PBPK model for amikacin and extended it to neonates to evaluate the achievement of pharmacodynamic targets. This model only predicted plasma drug concentration for 15 mg/kg q24h dosing in neonates aged 0–28 days.

The amikacin PBPK model developed in the current study provided an in-depth evaluation of drug penetration in various tissues (blood, lung, heart, and skin) under dosing regimens of 7.5 mg/kg q12h and 15 mg/kg q12h. Compared to previous models, this model offers a more detailed prediction of drug distribution in tissues, providing new insights into drug distribution in critical infection sites such as the lungs and heart.

The current model shares foundational physiological modeling frameworks with existing PBPK models, including drug distribution between organs and metabolic pathways. These are fundamental building blocks of PBPK modeling and are thoroughly utilized in this study.

#### 2.4.2 Pediatric dosing regimens

The recommended IV dosing regimens of polymyxin-B were 1.5–2.5 mg/kg/day for both adults and pediatrics (FDA, 2011). A loading dose and higher maintenance dose of up to 3 mg/kg/day were often used in clinical practice when treating MDR bacterial infection. When MIC was less than 0.5 mg/L, the dosage of 1.5–3.0 mg/kg/day could achieve over 90% probability of target attainments (PTA) in pediatric patient (Wang et al., 2022).

Nephrotoxicity is the main factor to consider when amikacin is administered to patients. The recommended dosing regimen of amikacin for patients with normal renal function is 15 mg/kg/day once-daily or divided in two or three equal doses (Perez-Blanco et al., 2021). At this dose, the trough concentration is maintained at <10 μg/mL in majority of the patients to minimize toxicity. EUCAST has recently changed antimicrobial susceptibility endpoints and increased dosing recommendation up to 30 mg/kg/day (EUCAST, 2020a); no increased toxicity for amikacin administered at higher doses (25–30 mg/kg/day) than the standard 15 mg/kg/day has been shown in specific populations including severe sepsis and critically ill patients (Galvez et al., 2011; de Montmollin et al., 2014; Kato et al., 2017; Perez-Blanco et al., 2021; Frost et al., 2023).

The dosing regimen of sulbactam in pediatrics was utilized according to our previous study (Zhu et al., 2022a). Both amikacin and sulbactam in pediatrics were grouped by pediatric body weight assuming normal renal function. Table 2 shows the dosing regimens for polymyxin-B, amikacin and sulbactam used in the pediatric population.

**Table 2:** Dosing regimens of amikacin/sulbactam/polymyxin-B used in simulation by creatinine clearance and by body weight categories.

Category	Dosing regimens
*Creatinine clearance*	*Amikacin/sulbactam (adult)*
≥60 mL/min	15 mg/kg q24h/3 g q8h as continuous infusion
≥60 mL/min	30 mg/kg q24h/3 g q8h as continuous infusion
40–59 mL/min	15 mg/kg q36h/3 g q8h as 3 h infusion
40–59 mL/min	30 mg/kg q36h/3 g q8h as 3 h infusion
30–39 mL/min	15 mg/kg q48h/3.5 g q12h as 4 h infusion
30–39 mL/min	30 mg/kg q48h/3.5 g q12h as 4 h infusion
*Body weight*	*Amikacin/sulbactam (pediatric)*
≥40 kg	7.5 mg/kg q12h/1.5 g q6h as 3 h infusion
≥40 kg	15 mg/kg q12h/1.5 g q6h as 3 h infusion
<40 kg	7.5 mg/kg q12h/50 mg/kg q6h as 3 h infusion
<40 kg	15 mg/kg q12h/50 mg/kg q6h as 3 h infusion
*Adult*	*polymyxin-B (adult)*
All renal function	LD 2.5 mg/kg + 1.5 mg/kg q12h as 1 h infusion
All renal function	LD 2.0 mg/kg + 1.25 mg/kg q12h as 1 h infusion
*Child (2 to<18 years of age)*	*polymyxin-B (pediatric)*
Normal renal function	1.25 mg/kg q12h as 1 h infusion
*Infant (0 to<2 years of age)*	*polymyxin-B (pediatric)*
Normal renal function	2.0 mg/kg q12h as 1 h infusion

LD, loading dose.

### 2.5 Tissue drug concentrations and penetration rates

Drug concentrations in tissues including blood, lung, heart and skin for the three antibiotics were simulated and predict by PBPK model in both pediatric and adult populations. The penetration rate was determined by comparing the *C*_max_ and AUC of each tissue to that in the blood. To ensure the accuracy of our simulation results, we compared the results with those reported in the literature.

### 2.6 Pharmacodynamic indices and probability of target attainment

For antibiotic pharmacodynamics (PD), the effectiveness of a drug is closely related to its exposure at the infection site. Antibiotics are generally classified into three categories: time-dependent, concentration-dependent, and time-dependent with a long post-antibiotic effect (PAE) (Sy et al., 2016).

The bactericidal effect of time-dependent antibiotics depends on the duration of time the free drug concentration exceeds the minimum inhibitory concentration (MIC) of the bacteria. The PD index for these antibiotics is the percentage of time the drug concentration remains above MIC during the dosing interval (*f*T > MIC). The PD index of sulbactam is 40% *f*T > MIC against lung infection model of *A. baumannii* (Yokoyama et al., 2015). The effectiveness of these antibiotics relies on maintaining an effective concentration for a sufficient period to ensure antibacterial activity.

For time-dependent antibiotics with a long PAE, the PD index is the ratio of the free drug exposure over 24 h to MIC (*f*AUC_0–24_/MIC). The killing effect of polymyxin B is associated with *f*AUC_0–24_/MIC index. An *f*AUC/MIC range of 8.2–42.1 was associated with a 2 log kill in the lung infection model (Dudhani et al., 2010; Sandri et al., 2013a; Zhu et al., 2022b). Thus, *f*AUC/MIC ≥8.2 was selected as the PD index of polymyxin B against *A. baumannii*.

The antibacterial effect of concentration-dependent antibiotics is related to the level of the free drug concentration. The higher the concentration, the better the bactericidal effect. The PD index for these drugs can be the peak drug concentration (*C*_max_) to MIC ratio (*f*C_max_/MIC) or *f*AUC_0–24_/MIC. For amikacin, the *f*C_max_/MIC target value should be at least 8 for a satisfactory therapeutic effect, and *f*AUC/MIC ≥80 is also recommended (Moore et al., 1987; Rybak et al., 1999; Darlow et al., 2024).

In the case of monotherapy, the probability of target attainment (PTA) was defined as the proportion of the simulated concentration-time profiles that could achieve the target indices. For combination therapy, we have previously proposed that the joint PTA be the minimum of the individual PTAs of the antibiotics in the combination. A sufficient probability of success is when the MICs of each drug in the combination are associated with ≥90% PTA (Menegucci et al., 2019; Martins et al., 2021). The PD index and PTA analyses were carried out using user-defined functions in R (version 4.1.2).

## 3 Results

### 3.1 PBPK model qualification in adult and pediatric populations

**Figure 1:**
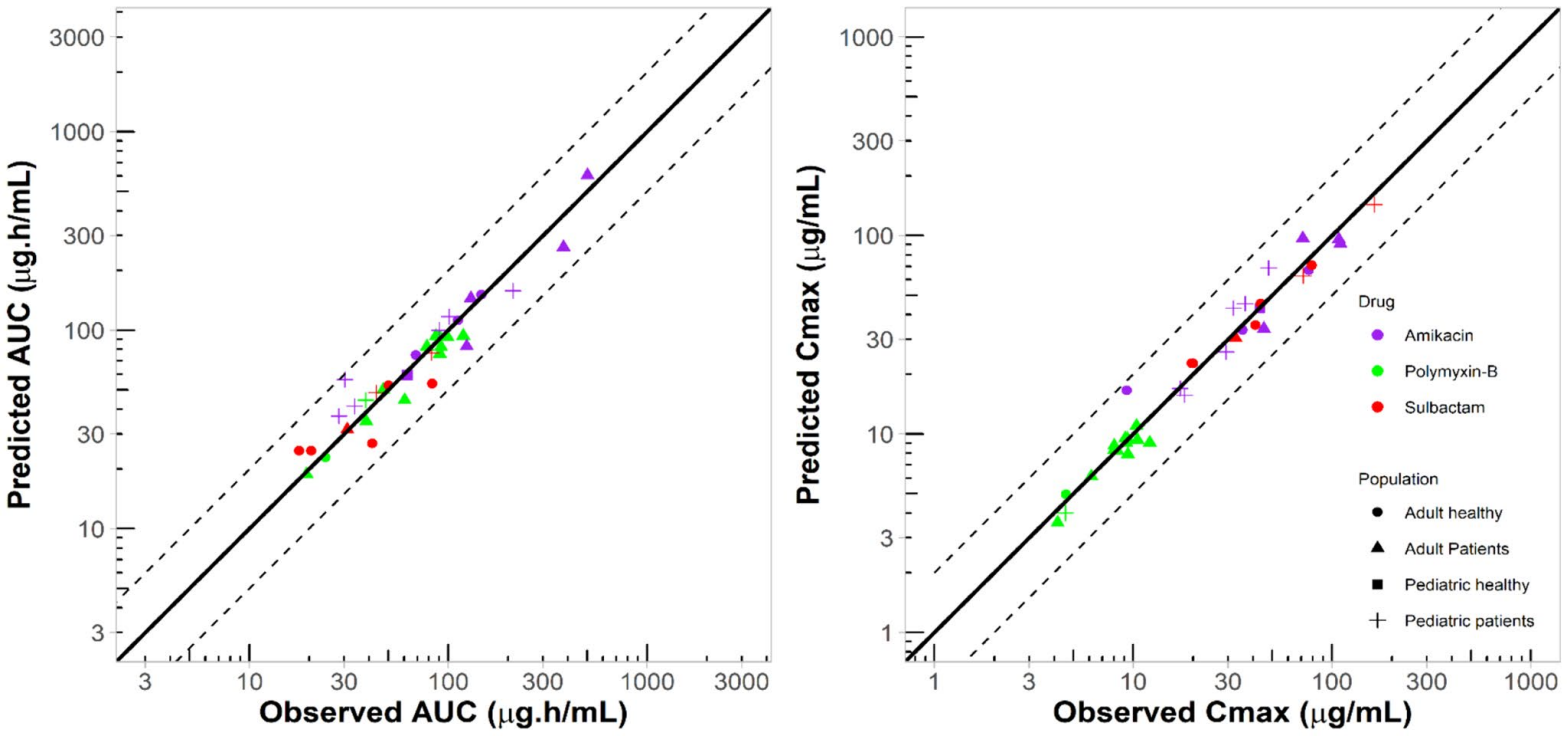
Comparison between simulated and observed exposure parameters from several studies in the literature for different populations. Solid lines represent line of unity; dashed lines represent two-fold difference.

The PBPK model development and evaluation for polymyxin-B, amikacin and sulbactam were sourced from 5, 10, and 4 reports containing 5, 11, and 6 populations, respectively; 17, 285, and 70 observations of polymyxin-B, amikacin and sulbactam PK profiles, respectively, from adults and pediatrics were utilized for model verification (Supplementary Tables S1–S3). The disease statuses of patients used in the development of PBPK models are listed in Supplementary Table S4. The collection of PK data used for PBPK model development came from diverse medical conditions. The observed data extracted from the literature for polymyxin-B, amikacin and sulbactam were within the 95% prediction interval of the corresponding simulations (Supplementary Figure S1). The two key exposure parameters, *C*_max_ and AUC, for adult and pediatric simulation were all within the MFE boundary of 0.5 to 2.0 compared to observed data (Figure 1).

### 3.2 Tissue exposures

We compared the PBPK model to the population PK model for adult dosing regimen by renal function and evaluated the interindividual variability in pharmacokinetic profiles (Supplementary Figures S2, S3). The interstitial fluid compartment is the site where we simulated representative tissue drug concentrations. The penetration rates (computed as AUC ratio or *C*_max_ ratio for tissue/plasma) from the simulation and the literature were compared to evaluate the accuracy of the model.

The penetration ratios of amikacin in heart, lung and skin were 1.10–1.24, 0.49–0.58, and 0.41–0.46, respectively (Tables 3, 4). The cardiac partition coefficient was adjusted to reflect higher exposure of amikacin in the heart. The literature reported values were as follows: heart tricuspid, 1.15–1.27; lung alveolar, 0.50; skin blister fluid, 0.54 (Lanao et al., 1983; Najmeddin et al., 2020; Shin et al., 2024). Sulbactam penetration rates in the heart, lung and skin were 0.50–0.51, 0.58–0.59 and 0.28–0.31, respectively (Tables 3, 4). The pulmonary penetration ratio was 52% following IV administration in healthy adult subjects (Rodvold et al., 2018). For other organs, the penetration ratios of sulbactam were not reported for humans.

**Table 3:** Simulated steady-state AUC_0–24h_ in various tissues and AUC ratio comparing tissue to plasma exposure of amikacin and sulbactam in adults and pediatrics 2 to <18 years of age.

Tissue	Amikacin AUC_Tissue_ (μg*h/mL)	Amikacin ratio	Sulbactam AUC_Tissue_ (μg*h/mL)	Sulbactam ratio
Adult CrCL ≥ 60 mL/min (15 mg/kg q24h/3 g q8h as continuous infusion)				
Plasma	179.6 ± 53.8	–	457 ± 105	–
Heart	196.0 ± 68.1	1.09	233 ± 52	0.51
Lung	89.7 ± 40.8	0.50	235 ± 52	0.52
Skin	73.7 ± 31.8	0.41	128 ± 35	0.28
Adult CrCL ≥ 60 mL/min (30 mg/kg q24h/3 g q8h as continuous infusion)				
Plasma	359.1 ± 107.7	–	457 ± 105	–
Heart	392.0 ± 136.3	1.09	233 ± 52	0.51
Lung	179.4 ± 81.6	0.50	235 ± 52	0.52
Skin	147.3 ± 63.7	0.41	128 ± 35	0.28
Adult CrCL 40 to 59 mL/min (15 mg/kg q36h/3 g q8h as 3 h infusion)				
Plasma	244.6 ± 57.2	–	771 ± 160	–
Heart	271.1 ± 83.9	1.11	386 ± 68	0.50
Lung	122.5 ± 48.5	0.50	395 ± 79	0.51
Skin	102.4 ± 35.2	0.42	224 ± 40	0.29
Adult CrCL 40 to 59 mL/min (30 mg/kg q36h/3 g q8h as 3 h infusion)				
Plasma	489.2 ± 114.4	–	771 ± 160	–
Heart	542.2 ± 167.8	1.11	386 ± 68	0.50
Lung	245.0 ± 97.0	0.50	395 ± 79	0.51
Skin	204.8 ± 70.4	0.42	224 ± 40	0.29
Adult CrCL 30 to 39 mL/min (15 mg/kg q48h/3.5 g q12h as 4 h infusion)				
Plasma	285.5 ± 60.2	–	719 ± 176	–
Heart	313.6 ± 89.7	1.10	367 ± 74	0.51
Lung	142.3 ± 56.5	0.50	370 ± 88	0.52
Skin	118.6 ± 40.1	0.42	208 ± 42	0.29
Adult CrCL 30 to 39 mL/min (30 mg/kg q48h/3.5 g q12h as 4 h infusion)				
Plasma	570.9 ± 120.3	–	719 ± 176	–
Heart	627.2 ± 179.5	1.11	367 ± 74	0.51
Lung	284.5 ± 112.9	0.50	370 ± 88	0.52
Skin	237.2 ± 80.3	0.42	208 ± 42	0.29
Pediatric ≥ 40 kg (7.5 mg/kg q12h/1.5 g q6h as 3 h infusion)				
Plasma	150.2 ± 41.1	–	381 ± 98	–
Heart	165.1 ± 54.6	1.10	194 ± 46	0.51
Lung	76.6 ± 33.4	0.51	196 ± 49	0.51
Skin	63.0 ± 24.3	0.42	110 ± 19	0.29
Pediatric ≥ 40 kg (15 mg/kg q12h/1.5 g q6h as 3 h infusion)				
Plasma	300.3 ± 82.2	–	381 ± 98	–
Heart	330.2 ± 109.3	1.10	194 ± 46	0.51
Lung	153.2 ± 66.7	0.51	196 ± 49	0.51
Skin	126.0 ± 48.6	0.42	110 ± 19	0.29
Pediatric < 40 kg (7.5 mg/kg q12h/50 mg/kg q6h as 3 h infusion)				
Plasma	120.1 ± 33.1	–	488 ± 128	–
Heart	132.9 ± 43.2	1.11	249 ± 61	0.51
Lung	59.5 ± 26.0	0.50	251 ± 65	0.51
Skin	49.9 ± 20.3	0.42	151 ± 36	0.31
Pediatric < 40 kg (15 mg/kg q12h/50 mg/kg q6h as 3 h infusion)				
Plasma	240.3 ± 66.3	–	488 ± 128	–
Heart	265.8 ± 86.5	1.11	249 ± 61	0.51
Lung	119.1 ± 52.1	0.50	251 ± 65	0.51
Skin	99.8 ± 40.7	0.42	151 ± 36	0.31

CrCL, creatinine clearance.

**Table 4:** Simulated steady-state *C*_max_ in various tissues and *C*_max_ ratio comparing tissue to plasma exposure of amikacin and sulbactam in adults and pediatrics 2 to <18 years of age.

Tissue	Amikacin *C*_max_ (μg/mL)	Amikacin ratio	Sulbactam *C*_max_ (μg/mL)	Sulbactam ratio
Adult CrCL ≥ 60 mL/min (15 mg/kg q24h/3 g q8h as continuous infusion)				
Plasma	51.2 ± 11.6	–	20.1 ± 4.8	–
Heart	61.8 ± 16.3	1.22	10.2 ± 2.1	0.51
Lung	29.3 ± 11.1	0.58	10.3 ± 2.4	0.51
Skin	22.6 ± 7.5	0.45	6.43 ± 1.54	0.32
Adult CrCL ≥ 60 mL/min (30 mg/kg q24h/3 g q8h as continuous infusion)				
Plasma	102.5 ± 23.2	–	20.1 ± 4.8	–
Heart	123.6 ± 32.6	1.22	10.2 ± 2.1	0.51
Lung	58.6 ± 22.2	0.58	10.3 ± 2.4	0.51
Skin	45.1 ± 15.0	0.45	6.43 ± 1.54	0.32
Adult CrCL 40 to 59 mL/min (15 mg/kg q36h/3 g q8h as 3 h infusion)				
Plasma	55.1 ± 13.4	–	69.5 ± 11.8	–
Heart	66.8 ± 19.0	1.22	35.7 ± 5.9	0.51
Lung	31.2 ± 11.8	0.57	35.7 ± 5.7	0.51
Skin	24.5 ± 7.6	0.45	18.8 ± 2.8	0.27
Adult CrCL 40 to 59 mL/min (30 mg/kg q36h/3 g q8h as 3 h infusion)				
Plasma	110.3 ± 26.8	–	69.5 ± 11.8	–
Heart	133.6 ± 38.1	1.22	35.7 ± 5.9	0.51
Lung	62.4 ± 23.6	0.57	35.7 ± 5.7	0.51
Skin	49.1 ± 15.3	0.45	18.8 ± 2.8	0.27
Adult CrCL 30 to 39 mL/min (15 mg/kg q48h/3.5 g q12h as 4 h infusion)				
Plasma	56.6 ± 13.2	–	74.4 ± 14.1	–
Heart	67.9 ± 18.6	1.21	38.6 ± 7.1	0.51
Lung	31.9 ± 12.3	0.57	38.5 ± 7.0	0.52
Skin	25.0 ± 8.0	0.45	20.1 ± 3.3	0.27
Adult CrCL 30 to 39 mL/min (30 mg/kg q48h/3.5 g q12h as 4 h infusion)				
Plasma	113.2 ± 26.5	–	74.4 ± 14.1	–
Heart	135.9 ± 37.2	1.21	38.6 ± 7.1	0.51
Lung	63.7 ± 24.7	0.57	38.5 ± 7.0	0.52
Skin	50.0 ± 16.0	0.45	20.1 ± 3.3	0.27
Pediatric ≥ 40 kg (7.5 mg/kg q12h/1.5 g q6h as 3 h infusion)				
Plasma	25.2 ± 5.6	–	29.2 ± 6.6	–
Heart	30.1 ± 7.9	1.21	15.1 ± 3.1	0.51
Lung	14.3 ± 5.3	0.57	15.0 ± 3.3	0.51
Skin	11.4 ± 3.7	0.46	8.47 ± 1.9	0.29
Pediatric ≥ 40 kg (15 mg/kg q12h/1.5 g q6h as 3 h infusion)				
Plasma	50.4 ± 11.3	–	29.2 ± 6.6	–
Heart	60.2 ± 15.8	1.21	15.1 ± 3.1	0.51
Lung	28.6 ± 10.6	0.57	15.0 ± 3.3	0.51
Skin	22.8 ± 7.4	0.46	8.47 ± 1.9	0.29
Pediatric < 40 kg (7.5 mg/kg q12h/50 mg/kg q6h as 3 h infusion)				
Plasma	21.0 ± 4.2	–	38.3 ± 8.8	–
Heart	25.7 ± 6.2	1.23	19.2 ± 5.1	0.50
Lung	11.8 ± 4.4	0.57	19.7 ± 4.5	0.51
Skin	9.7 ± 3.3	0.47	11.5 ± 2.5	0.30
Pediatric < 40 kg (15 mg/kg q12h/50 mg/kg q6h as 3 h infusion)				
Plasma	42.1 ± 8.5	–	38.3 ± 8.8	–
Heart	51.4 ± 12.3	1.23	19.2 ± 5.1	0.50
Lung	23.6 ± 8.8	0.57	19.7 ± 4.5	0.51
Skin	19.4 ± 6.6	0.47	11.5 ± 2.5	0.30

CrCL, creatinine clearance.

For polymyxin-B, these values were 0.980–1.05, 2.80–2.96, and 1.46–1.53 (Tables 5, 6). The penetration ratios of polymyxin-B in the heart and lungs were 1.03–1.12 and 1.93–3.38 in the rat model, respectively (Manchandani et al., 2016).

**Table 5:** Simulated steady-state AUC_0–24h_ in various tissues and AUC ratio comparing tissue to plasma exposure of polymyxin-B in adults and pediatrics 2 to <18 years of age.

Tissue	Polymyxin-B AUC_tissue_ (μg*h/mL)	Polymyxin-B ratio
Adult all renal function LD 2.5 mg/kg + 1.5 mg/kg q12h as 1 h infusion		
Plasma	96.8 ± 24.0	–
Heart	96.9 ± 41.6	0.990
Lung	293 ± 125	3.05
Skin	148 ± 56	1.55
Adult all renal function LD 2.0 mg/kg + 1.25 mg/kg q12h as 1 h infusion		
Plasma	83.2 ± 20.2	–
Heart	84.7 ± 29.7	1.02
Lung	236 ± 109	2.88
Skin	131 ± 54	1.57
Child all renal function (2 to<18 years of age) 1.25 mg/kg q12h as 1 h infusion		
Plasma	47.1 ± 13.2	–
Heart	49.2 ± 20.4	1.05
Lung	135 ± 63	2.88
Skin	73.4 ± 30.9	1.55
Infant all renal function (0 to<2 years of age) 2.0 mg/kg q12h as 1 h infusion		
Plasma	64.8 ± 18.2	–
Heart	66.3 ± 27.4	1.02
Lung	186 ± 89.3	2.87
Skin	99.8 ± 41.9	1.54

LD, loading dose.

**Table 6:** Simulated steady-state *C*_max_ in various tissues and *C*_max_ ratio comparing tissue to plasma exposure of polymyxin-B in adults and pediatrics 2 to <18 years of age.

Tissue	Polymyxin-B *C*_max_ (μg/mL)	Polymyxin-B ratio
Adult all renal function LD 2.5 mg/kg + 1.5 mg/kg q12h as 1 h infusion		
Plasma	10.9 ± 1.2	–
Heart	11.7 ± 4.0	1.06
Lung	36.6 ± 13.7	3.36
Skin	16.5 ± 4.5	1.52
Adult all renal function LD 2.0 mg/kg + 1.25 mg/kg q12h as 1 h infusion		
Plasma	9.20 ± 1.03	–
Heart	10.1 ± 2.8	1.10
Lung	28.9 ± 11.9	3.18
Skin	14.0 ± 3.7	1.54
Child all renal function (2 to<18 years of age) 1.25 mg/kg q12h as 1 h infusion		
Plasma	5.22 ± 0.82	–
Heart	5.58 ± 1.90	1.07
Lung	15.5 ± 6.2	2.99
Skin	8.14 ± 2.68	1.56
Infant all renal function (0 to<2 years of age) 2.0 mg/kg q12h as 1 h infusion		
Plasma	7.31 ± 1.06	–
Heart	7.74 ± 2.58	1.06
Lung	22.2 ± 8.8	3.05
Skin	11.3 ± 3.6	1.55

LD, loading dose.

The standard deviation of the partition coefficients of tissue/plasma for polymyxin-B and amikacin in PK-Sim were adjusted and the resulting coefficient of variation (CV) of tissue *C*_max_ and AUC were also compared to that reported in the literature. For amikacin, the CV% of simulated *C*_max_ in the blood ranged from 20.6 to 23.7%, which was consistent with the reported CV% or *C*_max_ of 23.0% (Najmeddin et al., 2020). Since amikacin exposure in the heart mirrors that in the blood, the CV% in the heart tissue ranged from 24.1 to 28.3%, similar to those in the blood. In the lung and skin, the CV% ranged from 37.2 to 38.6% and 30.9 to 34.3%, respectively. These values were also consistent with the reported CV% or *C*_max_, which were 38.6 and 38.8%, respectively in the same tissues (Lanao et al., 1983; Bayer et al., 1988). There are no reported exposure parameters of polymyxin-B in human tissues or body fluids other than the blood. Instead, we compared the CV values to those reported in the rat; the CV of polymyxin-B in the rat’s heart and lung were 31.6–51.2% and 30.8–75.3%, respectively (Manchandani et al., 2016). The corresponding CV values from our PBPK simulations in humans were 31.3–33.6% and 33.0–42.1% for the same respective organs. As for sulbactam, the variability due to PBPK simulation in the lung was 14.7–22.9%, which is close to the corresponding CV of 29.6% from the literature (Rodvold et al., 2018).

### 3.3 Probability of target attainment (PTA) for pediatric dosing regimens

**Figure 2:**
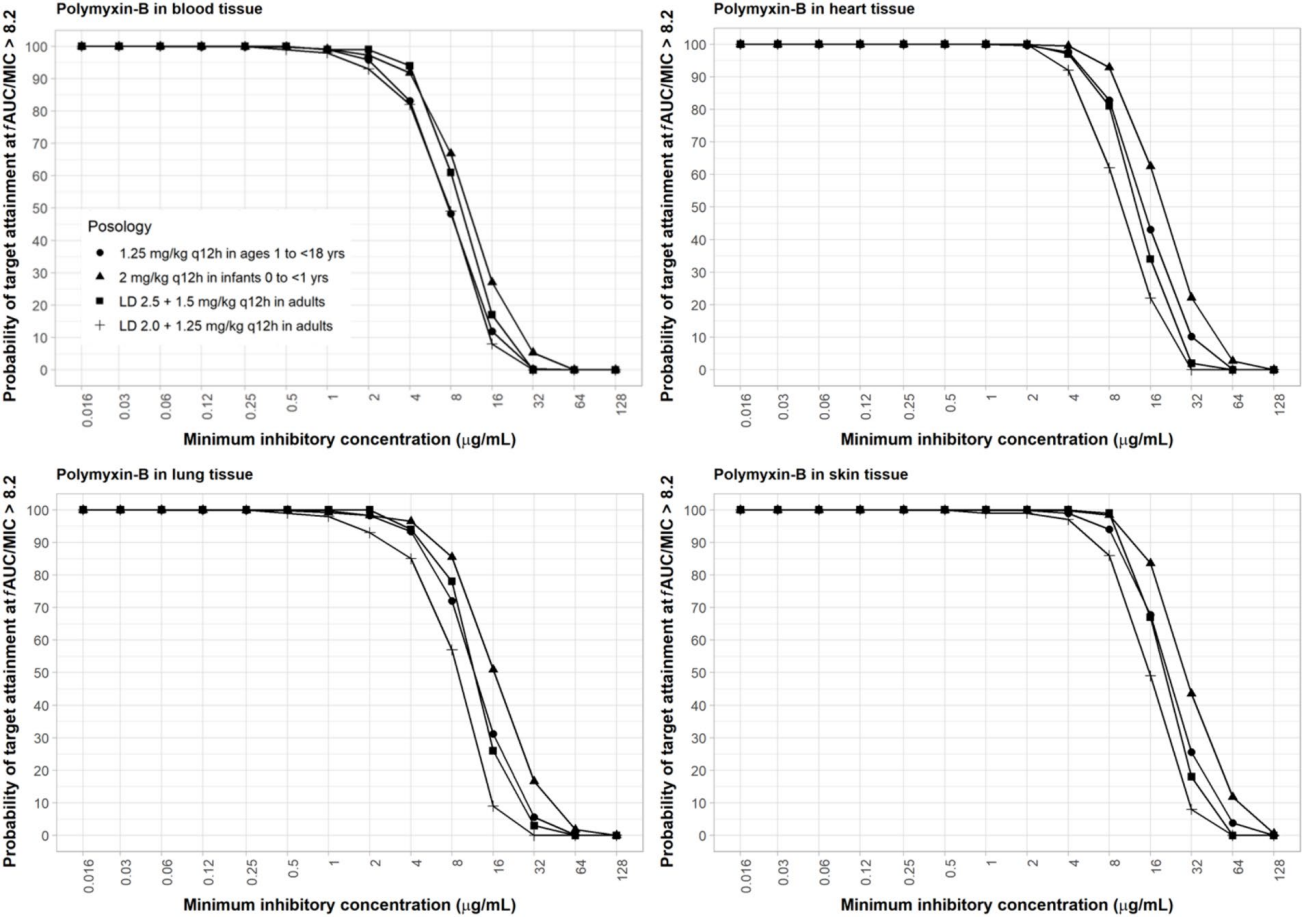
Probability of target attainment of polymyxin-B PD index of *f*AUC/MIC of at least 8.2 in the blood, lung, heart, and skin for adult and pediatric dosing regimens.

The interpretive criteria for polymyxin-B against *A. baumannii* are the following: intermediate, MIC ≤2 μg/mL; and resistant, MIC ≥4 μg/mL (Satlin et al., 2020; CLSI, 2024). We assumed that the target pharmacodynamic index for polymyxin-B (i.e., *f*AUC/MIC≥8.2) is the same for the blood and other tissues. The IV administration of polymyxin-B in the pediatric population was simulated to evaluate drug concentrations in pediatric tissues. The results in Figure 2 showed that in the blood, dosing regimens consisting of a loading dose (LD) 2.5 mg/kg plus maintenance dose (MD) 1.5 mg/kg q12h in adults and 2 mg/kg q12h in pediatrics (0 to <2 years old) were able to achieve more than 90% PTA for an MIC of ≤4 μg/mL whereas LD 2.0 + 1.25 mg/kg q12h in adults and 1.25 mg/kg q12h in pediatric (2 to <18 years old) achieved similar results for ≤2 μg/mL MIC. In cardiac tissues, all dosing regimens achieved more than 90% PTA at ≤4 μg/mL MIC, and pediatric (0 to <2 years old) 2 mg/kg q12h achieved more than 90% PTA at ≤8 μg/mL MIC. In the lung, an adequate coverage (PTA ≥85%) was achieved at ≤4 μg/mL MIC for all listed regimens. In the skin, all dosing regiments achieved >85% PTA at ≤8 μg/mL MIC. Polymyxin-B adult and pediatric dosing regimens have comparable exposures.

Amikacin approved dosing regimen is 15 mg/kg once-daily or 7.5 mg/kg twice-daily. A revised dosing of 25–30 mg/kg once daily has been recommended by EUCAST, given the risk of sub-therapeutic drug concentrations with the 15 mg/kg once daily dosing against pathogens with high MIC value (4–16 mg/L) (EUCAST, 2020a). EUCAST susceptibility breakpoints are 8 mg/L and 16 mg/L for Enterobacterales and *Pseudomonas* sp., respectively (EUCAST, 2020b). Both the approved and revised dosing regimens were evaluated. PBPK simulations of both adult and pediatric dosing regimens indicated that drug exposure was highest in the heart, followed by the blood, lungs, and skin. A reason for a slightly higher exposure of amikacin in the heart compared to plasma is that lower blood flow and pressure that are usually present in the cardiac tricuspid valve tissue compared to the aorta can result in longer blood retention time, resulting in a higher drug exposure (McColm and Ryan, 1985; Bayer et al., 1988).

**Figure 3:**
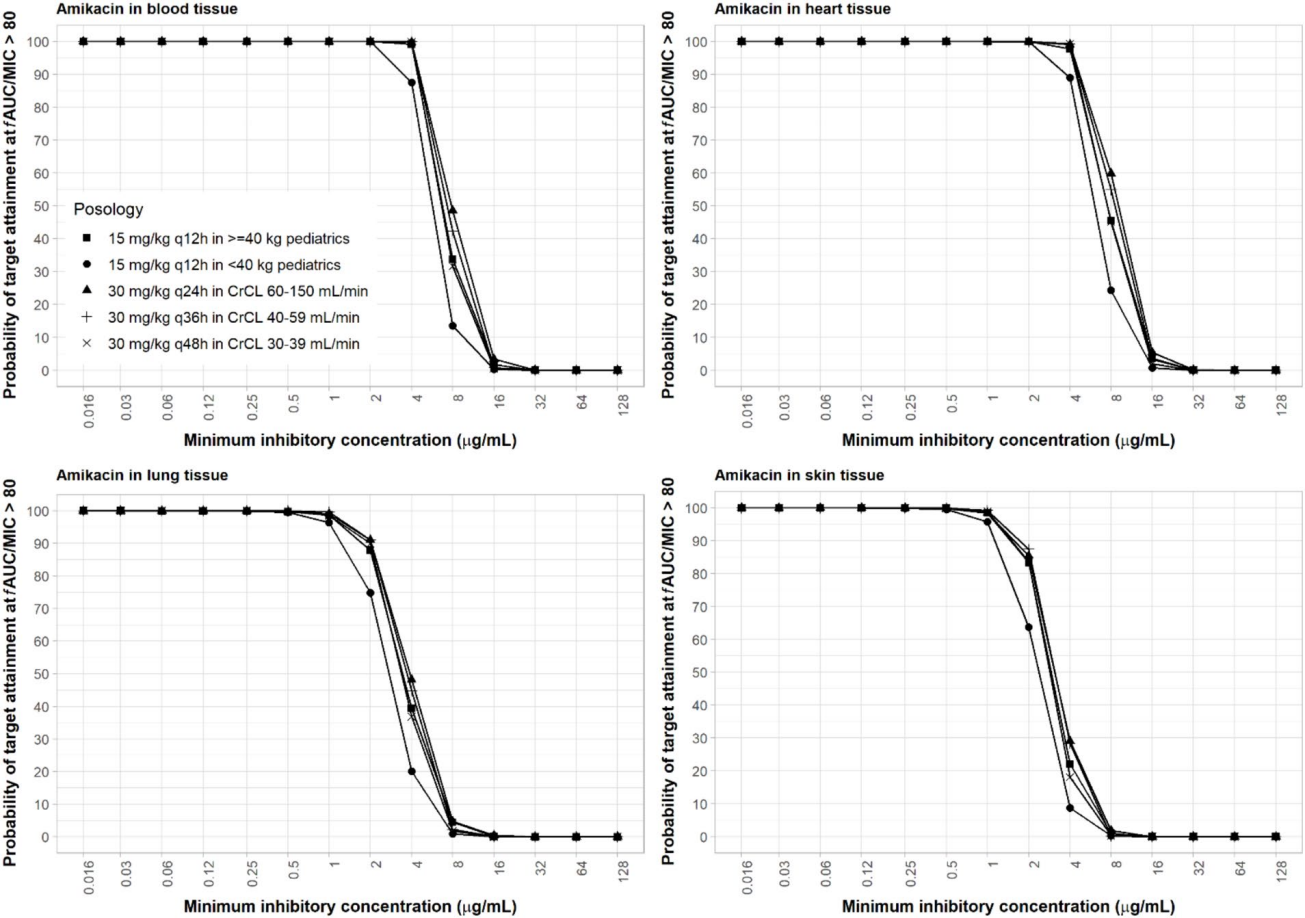
Probability of target attainment of amikacin PD index of *f*AUC/MIC ratio of at least 80 in the blood, lung, heart, and skin for dosing regimens in pediatrics (15 mg/kg) and adults (30 mg/kg).

For amikacin, two relevant PD target indices are *f*C_max_/MIC ≥8 and *f*AUC/MIC ≥80. The *f*AUC/MIC target at 80–90 is believed to be a more robust alternative and may be more suitable for critically ill patients with high bacterial burden infections such as nosocomial pneumonia (Bland et al., 2018; De winter et al., 2018; Perez-Blanco et al., 2021). Due to differences in dosing frequencies of amikacin between adults with renal insufficiencies and pediatrics, we used these two target PD indices for amikacin in our computation of PTA for a more thorough assessment. With the high dose of amikacin, at least 90% PTA was achieved in blood with an MIC of ≤16 μg/mL using ≥8 *f*C_max_/MIC (Supplementary Figure S5) and ≤4 μg/mL using ≥80 *f*AUC/MIC (Figure 3). The 15 mg/kg adult dosing regimens only attained half the MIC of the high dose (Supplementary Figures S6, S7). The 15 mg/kg twice-daily in pediatrics resulted in similar PTA as that of the adult of the same total daily dose using target AUC PD index (Figure 3) but lower PTA if PD index based on *C*_max_ was used (Supplementary Figure S6), since the twice-daily regimen resulted in only half the *C*_max_ as the once-daily regimen with the same total daily dose. As amikacin exposure in the lung and skin is lower than that in the blood, sufficient coverage can be achieved at ≤2 and ≤1 μg/mL MIC in the lung and skin, respectively (Figure 3) for the adult high dose with the AUC PD index. With the *C*_max_ PD index, sufficient coverage can be achieved at a two-fold higher MIC values. Due to low permeability of amikacin in the skin, skin infections are difficult to treat with intravenous amikacin administration.

**Figure 4:**
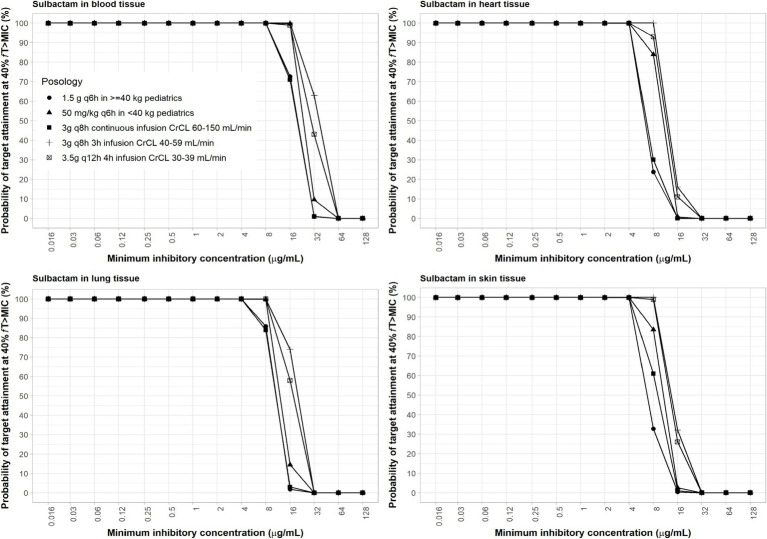
Probability of target attainment of sulbactam PD index of *f*T > MIC of at least 60% in the blood, lung, heart, and skin for adult and pediatric dosing regimens.

The interpretive criteria for *Acinetobacter* spp. susceptibility to sulbactam treatment is ≤4 μg/mL for susceptible and 8 μg/mL for intermediate (CLSI, 2024). Sulbactam pediatric regimen of 40 mg/kg q6h as 3-h infusion was selected for a body weight <40 kg. Previous studies have shown that sulbactam adult regimens against *A. baumannii* at 8 μg/mL MIC, using 40% *f*T > MIC as PD index, can reach ≥90% PTA (Yokoyama et al., 2015). The calculations assumed a plasma protein binding of 5% and no protein binding in other tissues (Yokoyama et al., 2014). Sulbactam was able to achieve ≥90% PTA in all age groups of pediatric patients with an MIC of ≤8 μg/mL using the administration schedule listed in Table 2. At 4 μg/mL MIC, coverage in the lung, skin, and heart were adequate based on the proposed dosing regimens of sulbactam (Figure 4).

In the lung, the MIC values for amikacin, polymyxin-B and sulbactam combination to sufficiently achieve satisfactory joint PTA are 2/4/4 μg/mL.

## 4 Discussion

The predicted antibiotic exposures using PBPK models and target achievement in major tissues associated with common infections including the heart, lungs, and skin can be used to infer efficacy at the site of infection. Often actual drug exposures in these commonly infected sites are lacking, especially drug concentrations in pediatric tissues and organs; pediatric data are more difficult to obtain than in adults (Kafetzis et al., 1979; Schaad et al., 1986; Xu et al., 2022). We established and validated PBPK models in adults and extrapolated them to the pediatric population to evaluate the therapeutic effects of these antibiotics at the sites of infection. The application of the PBPK model to determine site-specific drug concentrations assumes that PD targets and thresholds associated with microbial outcomes are appropriate not only for their assessment in the blood, but also in the infected tissues.

The clinical efficacy of antimicrobial combination is often inferred from case reports. The combination of polymyxin-B and amikacin has been shown to be effective against blood infections caused by *Klebsiella pneumoniae* and offered a survival benefit (Cleary et al., 1979). This combination therapy has not been studied in a randomized clinical trial. Polymyxin-B combined with cefoperazone/sulbactam successfully cured posterior ventriculitis caused by extensively resistant *A. baumannii* in a child. In another case, a patient presented with pneumonia-related multiple organ dysfunction syndrome due to MDR *P. aeruginosa* and *A. baumannii* was successfully treated with polymyxin-B/amikacin combination (Wang et al., 2021; Yang et al., 2021).

The combination strategy can sensitize MDR microorganisms to the drug concentrations produced using the approved dosing regimens. In our previous survey of extremely drug-resistant *A. baumannii* clinical isolates, the MIC_50_ and MIC_90_ for amikacin/polymyxin-B/sulbactam combination were 1/4/4 and 8/4/4 μg/mL, respectively (Zhu et al., 2022a). Based on the PTA evaluation for this combination in the blood, lung, heart and skin, sufficient coverage can be achieved for the MIC_50_ of the surveyed collection. Amikacin dosing regimens, on the other hand, would not provide sufficient coverage against MIC_90_ of the collection based on the stricter PD index using *f*AUC/MIC >80. However, the *f*C_max_/MIC >8 criteria can be achieved with the high dose of 30 mg/kg/day in the blood, heart and lung, but not the skin.

Our analysis indicated that drug exposures for this combination in the tissues or organs evaluated were effective against at least 50% of extremely drug resistant *A. baumannii* clinical isolates. The inference for clinical efficacy using PTA did not include the antibacterial effect of human immunity. In the *in vivo* infection model used to define target PD indices, neutropenia was induced in mice prior to infecting the animal (Sy and Derendorf, 2014). The methodology to derive PD indices reflects the worst-case scenario in immunocompromised individuals. The immune system will play an important role against MDR infections because it does not distinguish between resistant and sensitive bacteria (Rayner et al., 2021).

The variability in antibiotic permeability across different tissues led to differences in the PTA results. Amikacin, as an aminoglycoside, is a hydrophilic molecule with low tissue permeability. Passive diffusion across endothelial cells of capillaries requires drugs to be lipophilic, which may result in low amikacin exposure in the lungs and skin (Honeybourne, 1994; Najmeddin et al., 2020). However, amikacin is commonly used in the clinic for the treatment of acute exacerbations in patients with cystic fibrosis (Kiem and Schentag, 2008), which may be attributed to inflammation-induced lung endothelial damage, affecting alveolar epithelial permeability, thereby enhancing amikacin penetration and distribution into the lungs (Lamer et al., 1993). Although it can achieve high therapeutic concentrations in the lungs, amikacin clearance from the lungs is affected by its exchange in the blood.

Polymyxin B is a cationic polypeptide antibiotic, and its large molecular characteristics restrict its distribution, metabolism, and excretion after intravenous injection (Avedissian et al., 2019). Our predictions for lung exposure were consistent with the observed drug concentrations reported (Manchandani et al., 2016). The 6-h sample of polymyxin in the lung tissues was approximately 2-fold the serum drug concentration, indicating accumulation of polymyxin in the lung over time (Manchandani et al., 2016), whereas polymyxin exposure in the epithelial lining fluid in mice was previously shown to be lower than that in the serum (He et al., 2010). There is a high degree of variability in the literature on polymyxin lung penetration.

The current guidelines for polymyxins recommends combination therapy, since it is not possible to increase the daily doses beyond the recommended limit of 2.5 mg/kg loading dose and 1.5 mg/kg q12h maintenance dose (Tsuji et al., 2019). Lung infection model indicated lower efficacy than thigh infection model (Cheah et al., 2015). With increasing polymyxin resistance, the strategy to optimize polymyxin therapeutics could also include inhalation, in the case of lung infection (Zhu et al., 2022a).

While our PBPK model offers valuable insights into the pharmacokinetics of drug combinations in pediatric patients, particularly in predicting drug concentrations across various tissues, including the lungs, we must acknowledge the inherent uncertainties and limitations of these predictions. The complexity and variability of lung physiology, the heterogeneity in drug distribution, and the lack of comprehensive clinical data to fully validate these predictions present many challenges toward an accurate prediction of lung polymyxin concentrations.

Dosing guideline of antibiotics in pediatrics should be based on antimicrobial susceptibility determination. We do not recommend deviating from the recommended clinical dosages. Of the three antibiotics, only amikacin has a standard dose and a high dose, due to recent changes in consensus guidelines. Decision to use combination therapy should be based on whether the MIC for the combination can be sufficiently covered by the joint PTA.

Strategies for antibiotic combination use need to consider whether there is a potential risk of an enhanced toxicity. Consequently, the duration of treatment could be limited by adverse events. The polymyxin-B/amikacin/sulbactam triple combination has the potential for nephrotoxicity and other adverse effects. Polymyxin-B undergoes renal reabsorption through tubular cells while amikacin and sulbactam are primarily eliminated by the kidney. We chose polymyxin-B and sulbactam to be combined with amikacin because the renal liability of polymyxin-B is considerably less compared to colistin (Zavascki and Nation, 2017). The addition of sulbactam to the combination is based on a matched cohort study showing low potential of sulbactam to induce acute kidney injury when used as a partnering *β*-lactamase inhibitor to piperacillin (Rutter and Burgess, 2017). While amikacin is known to cause nephrotoxicity (Kaynar et al., 2007), the amikacin/polymyxin-B combination in a case report did not result in acute kidney injury even though this combination was administered for the duration of 30 days; the patient’s follow-up serum creatinine was 75 μmol/L which indicated no evidence of acute renal impairment (Wang et al., 2021).

Several limitations of the current approach are identified. The lack of actual tissue drug concentration data in human tissues limits our ability to verify the simulation of drug concentration. The complexity of organ tissue structure also affects the accuracy of drug concentration simulation; the microanatomy of tissue can lead to concentration gradients between compartments. The tissue drug concentration simulated in this study comes from interstitial space and may not necessarily represent the microspace where bacteria proliferate.

In summary, this study explored the use of PBPK models to predict drug exposure in several potential sites of infection in the pediatric population, and assessed whether exposure could achieve the desired target achievement rate. The results of the study have yet to be confirmed in clinical trials. Antibiotic combination offers a potential treatment option against tissue infections caused by drug-resistant bacteria, which are increasingly threatening human health. At a time when new antibiotics are scarce, effective antibiotic combination therapy has practical implications for addressing the pressing problem of drug resistance. PBPK and other modeling methods to predict and simulate the amount of antibacterial drug exposure in tissue is a practical approach to overcome the difficulty of obtaining tissue drug concentrations in pediatric population.

## Data Availability

The original contributions presented in the study are included in the article/Supplementary material, further inquiries can be directed to the corresponding authors.

## Footnotes

^1^https://go.drugbank.com/
