# Supplementary material for: Prediction of tissue exposures of polymyxin-B, amikacin and sulbactam using physiologically-based pharmacokinetic modeling
Source: Front Microbiol. 2024 Oct 7;15:1435906. doi: 10.3389/fmicb.2024.1435906 (PMC11491386; doi:10.3389/fmicb.2024.1435906)
Supplement: Supplementary file 1 [file Data_Sheet_1.docx]

**Supplementary Material for**

**Prediction of tissue exposures of polymyxin-B, amikacin and sulbactam using physiologically based pharmacokinetic modeling**

Mengyuan Wu^1#^, Kun Feng^2#^, Xiao Wu^4^, Shixing Zhu^1^, Frederico S. Martins^3^, Chang Liu^2^, Mingming Yu^1^, Zhihua Lv^1*^, Meixing Yan^2*^, Sherwin K. B. Sy^5*^

**1** School of Medicine and Pharmacy, Ocean University of China, Qingdao 266003, PR China

**2** Women and Children Hospital, Qingdao University, Qingdao 266034, China

**3** Department of Clinical and Toxicological Analysis, Faculty of Pharmaceutical Sciences, Universidade de São Paulo, São Paulo 05508-000, Brazil

**4** Qingdao Central Hospital, University of Health and Rehabilitation Sciences, Qingdao 266042, China

**5** Department of Statistics, Universidade Estadual de Maringá, Maringá, Paraná 87020-900, Brazil

# These authors contributed equally to this work and are co-first authors.

**Table S1:** Population characteristics, dosing information, observed and PBPK-predicted pharmacokinetic parameters of polymyxin-B used in the development and performance verification of polymyxin-B PBPK model

| **Population** | **Age years** | **N** | **Bodyweight (kg)** | **Average CLcr**  **(mL/min/1.73 m^2^)** | **Dose** | **AUC_0-last_** | **AUC_obs_** | **C_max,obs_** | **AUC_pred_** | **C_max,pred_** | **MFE_AUC_** | **MFE_Cmax_** | **Reference** |
| --- | --- | --- | --- | --- | --- | --- | --- | --- | --- | --- | --- | --- | --- |
| Adult Patients | 80 | 1 | 60.0 | <10 | 0.83 mg/kg IV q48h 1h | AUC_0-12h_ | 99.1 | 10.5 | 92.0 | 9.35 | 0.928 | 0.890 | [1] |
| Adult Patients | 52 | 1 | 80.0 | 83 | 1.25 mg/kg IV q12h 1h | AUC_0-12h_ | 119 | 12.1 | 93.8 | 9.04 | 0.788 | 0.747 |  |
| Adult Patients | 42 | 1 | 50.0 | 98 | 1.0 mg/kg IV q12h 1h | AUC_0-6h_ | 78.2 | 8.01 | 82.8 | 8.30 | 1.06 | 1.04 |  |
| Adult Patients | 72 | 1 | 75.0 | 60 | 1.0 mg/kg IV q12h 1h | AUC_0-12h_ | 78.2 | 8.02 | 82.4 | 8.72 | 1.05 | 1.09 |  |
| Adult Patients | 86 | 1 | 70.0 | 34 | 1.0 mg/kg IV q12h 1h | AUC_0-12h_ | 92.0 | 9.35 | 82.5 | 9.47 | 0.897 | 1.01 |  |
| Adult Patients | 72 | 1 | 67.0 | 26 | 1.5 mg/kg IV q48h 1h | AUC_0-12h_ | 90.7 | 9.40 | 76.0 | 7.90 | 0.838 | 0.840 |  |
| Adult Patients | 65 | 1 | 60.0 | <10 | 0.5 mg/kg IV q48h 1h | AUC_0-12h_ | 86.7 | 9.10 | 93.5 | 9.51 | 1.08 | 1.05 |  |
| Adult Patients | 48 | 1 | 68.0 | 246 | 1.0 mg/kg IV q12h 1h | AUC_0-12h_ | 90.1 | 9.22 | 93.0 | 9.03 | 1.03 | 0.979 |  |
| Adult Patients | -- | 1 | -- | Normal renal function | 1.25 mg/kg IV 1h | AUC_0-12h_ | 60.3 | 6.15 | 44.4 | 6.11 | 0.736 | 0.993 | [2] |
| Adult Patients | -- | 1 | -- | Normal renal function | 100 mg q12h IV 1h | AUC_0-12h_ | 38.7 | 8.33 | 34.7 | 8.20 | 0.897 | 0.984 | [3] |
| Adult Patients | -- | 1 | -- | Normal renal function | 125 mg q12h IV 1h | AUC_0-12h_ | 47.1 | 10.4 | 50.2 | 11.0 | 1.07 | 1.06 |  |
| Adult HV | -- | 5 | -- | Normal renal function | 0.75 mg/kg IV 1 h | AUC_0-24h_ | 24.1 | 4.59 | 22.9 | 4.96 | 0.950 | 1.08 | [4] |
| Pediatric patients | 7 | 1 | -- | 69.7 | 1.0 mg/kg IV q12 h 1 h | AUC_0-24h_ | 38.4 | 4.56 | 44.4 | 4.00 | 1.16 | 0.877 | [5] |

HV, Healthy Volunteers; Num, number of subject; IV, intravenous; AUC, area under the curve; CLcr, creatinine clearance; Cmax, maximum drug concentration; MFE, mean fold error; obs, observed; pred, predicted

Units: Dose in mg; Age in years, AUC in µg*h/mL; Cmax in µg/mL**Table S2:** Population characteristics, dosing information, observed and PBPK-predicted pharmacokinetic parameters of amikacin used in the development and performance verification of amikacin PBPK model

| **Population** | **Age years** | **N** | **Bodyweight (kg)** | **Average CLcr**  **(mL/min/1.73 m^2^)** | **Dose** | **AUC_0-last_** | **AUC_obs_** | **C_max,obs_** | **AUC_pred_** | **C_max,pred_** | **MFE_AUC_** | **MFE_Cmax_** | **Reference** |
| --- | --- | --- | --- | --- | --- | --- | --- | --- | --- | --- | --- | --- | --- |
| Adult HV | 23 - 31 | 6 | 50 - 75 | Normal renal function | 7.5 mg/kg IV 0.5h | AUC_0-12h_ | 68.8 | 35.4 | 75.0 | 33.4 | 1.09 | 0.944 | [6] |
| Adult HV | 23 - 31 | 6 | 50 - 75 | Normal renal function | 15 mg/kg IV 0.5h | AUC_0-12h_ | 147 | 76.4 | 151 | 66.8 | 1.03 | 0.874 |  |
| Adult HV | NA | 5 | NA | Normal renal function | Load 3.33mg/kg IV 1h  maintain 1.0mg/kg q 1h | AUC_0-8h_ | 112 | 9.28 | 112 | 16.6 | 1.00 | 1.79 | [7] |
| Adult Patients | 44.4 ± 14.2 | 30 | 69.47 ± 9.57 | 69 ± 7.53 | 25 mg/kg IV 1h | AUC_0-24h_ | 380 | 71.2 | 262 | 96.8 | 0.689 | 1.36 | [8] |
| Adult Patients | 63 ± 13 | 74 | -- | 33 - 86 ml/min | 25 mg/kg IV 0.5h | AUC_0-24h_ | 502 | 110 | 604 | 90.8 | 1.20 | 0.825 | [9] |
| Adult Patients | 21 - 48 | 7 | 111.4 - 216.8 | Normal renal function | 1250 mg IV bolus | AUC_0-8h_ | 130 | 108 | 145 | 95.7 | 1.12 | 0.886 | [10] |
| Adult Patients | 26 - 66 | 12 | -- | Normal renal function | 7.5 mg/kg IV 0.5h | AUC_0-8h_ | 124 | 45.4 | 82.9 | 33.9 | 0.669 | 0.747 | [11] |
| Pediatric HV | 3 - 11 | 8 | 15 - 26 | 64 - 136 | 7.5 mg/kg IV bolus | AUC_0-6h_ | 62.3 | 43.4 | 59.2 | 42.9 | 0.950 | 0.988 | [12] |
| Pediatric patients | 1.1 - 11 | 10 | 9 - 35 | 5 - 79 | 7.5 mg/kg IV bolus | AUC_0-16h_ | 101 | 31.9 | 117 | 43.1 | 1.16 | 1.35 |  |
| Pediatric patients | 6 - 8 | 56 | 23.0 ±14.7 | Normal renal function | 20 mg/kg/day IV 0.5h | AUC_0-12h_ | 90.3 | 36.5 | 100 | 45.3 | 1.11 | 1.24 | [13] |
| Pediatric patients | 3 M - 14 | 25 | -- | Normal renal function | 20 mg/kg/day IV 0.5h | AUC_0-24h_ | 212 | 48.0 | 158 | 68.7 | 0.745 | 1.43 | [14] |
| Pediatric patients | 1 - 6 | 12 | -- | Normal renal function | 5 mg/kg IV q8h 0.5h | AUC_0-8h_ | 30.2 | 29.3 | 56.4 | 25.9 | 1.87 | 0.884 | [15] |
| Pediatric patients | 7 - 11 | 12 | -- | Normal renal function | 5 mg/kg IV q8h 1h | AUC_0-8h_ | 33.8 | 18.1 | 41.4 | 15.7 | 1.22 | 0.867 |  |
| Pediatric patients | 12 - 16 | 22 | -- | Normal renal function | 5 mg/kg IV q6h 1h | AUC_0-6h_ | 28.2 | 17.2 | 36.9 | 17.0 | 1.31 | 0.988 |  |

HV, healthy volunteers; Num, number of subject; IV, intravenous; LD, loading dose; MD, maintenance dosing; CBA, colistin base active; CMS, colistin methanesulfonate; AUC, area under the curve; Cmax, maximum drug concentration; MFE, mean fold error; obs, observed; pred, predicted; NA, Not avaliable

Units: Dose in mg; Age in years, AUC in µg*h/mL; Cmax in µg/mL

**Table S3:** Population characteristics, dosing information, observed and PBPK-predicted pharmacokinetic parameters of sulbactam used in the development and performance verification of sulbactam PBPK model

| **Population** | **Age years** | **N** | **Bodyweight (kg)** | **Average CLcr**  **(mL/min/1.73 m2)** | **Dose** | **AUC_0-last_** | **AUC_obs_** | **C_max,obs_** | **AUC_pred_** | **C_max,pred_** | **MFE_AUC_** | **MFE_Cmax_** | **Reference** |
| --- | --- | --- | --- | --- | --- | --- | --- | --- | --- | --- | --- | --- | --- |
| Adult HV | 22 - 36 | 10 | 62-72 | Normal renal function | 500 mg IV bolus | AUC_0-6h_ | 41.54 | 41.10 | 26.94 | 35.40 | 0.649 | 0.861 | [16] |
| Adult HV | 22 - 36 | 10 | 62-72 | Normal renal function | 1000 mg IV bolus | AUC_0-6h_ | 83.22 | 78.90 | 53.88 | 70.79 | 0.647 | 0.897 |  |
| Adult HV | NA | 4 | NA | Normal renal function | 500 mg IV 0.5h | AUC_0-4h_ | 20.48 | 20.00 | 24.70 | 22.67 | 1.21 | 1.13 | [17] |
| Adult HV | NA | 4 | NA | Normal renal function | 1000 mg IV 0.5h | AUC_0-6h_ | 50.09 | 43.70 | 52.71 | 45.35 | 1.05 | 1.04 |  |
| Adult HV | NA | 3 | NA | Normal renal function | MD 500 mg q6h IV 0.5h | AUC_0-8h_ | 17.78 | 19.70 | 24.70 | 22.67 | 1.39 | 1.15 |  |
| Adult Patients | 29-84 | 40 | NA | Normal renal function | 500 mg IV 0.5h | AUC_0-8h_ | 31.1 | 32.6 | 31.59 | 30.62 | 1.02 | 0.939 | [18] |
| Pediatric patients | 8.4 ± 3.5 | 6 | 27.3 ± 10.2 | Normal renal function | 12.5 mg/kg IV 3min | AUC_0-3h_ | 43.49 | 71.60 | 48.51 | 62.62 | 1.12 | 0.875 | [19] |
| Pediatric patients | 7.6 ± 3.8 | 7 | 31.9 ± 19.5 | Normal renal function | 25 mg/kg IV 3min | AUC_0-3h_ | 82.39 | 163.0 | 77.01 | 143.19 | 0.935 | 0.878 |  |

HV, healthy volunteers; Num, number of subject; IV, intravenous infusion; MD, multiple-dose; AUC, area under the curve; Cmax, maximum drug concentration; MFE, mean fold error; obs, observed; pred, predicted; NA, not avaliable

Units: Dose in mg; Age in years, AUC in µg*h/mL; Cmax in µg/mL

**Table S4:** Disease statuses of the patients used in the development of the PBPK model for polymyxin B, amikacin, and sulbactam.

| **Antibiotic Name** | **Population** | **N** | **Disease / Infection type** |
| --- | --- | --- | --- |
| Polymyxin-B | Adult Patients[1] | 8 | *P. aeruginosa/A. baumannii* infection |
|  | Adult Patients[2] | 1 | Critically ill patients (infection type unknown) |
|  | Adult Patients[3] | 2 | Critically ill patients (infection type unknown) |
|  | Adult HV[4] | 5 | / |
|  | Pediatric patients[5] | 1 | Carbapenem-resistant *Klebsiella pneumoniae* |
| Amikacin | Adult HV[6] | 12 | / |
|  | Adult HV[7] | 5 | / |
|  | Adult Patients[8] | 30 | Gram-negative septicemia |
|  | Adult Patients[9] | 74 | Severe sepsis/septic shock |
|  | Adult Patients[10] | 7 | Post-operative gastric bypass patients (without symptoms or signs of infection) |
|  | Adult Patients[11] | 12 | Patients with traumatic spinal cord injury who have peptic ulcers of grade III or higher |
|  | Pediatric HV[12] | 8 | / |
|  | Pediatric patients[12] | 10 | / |
|  | Pediatric patients[13] | 56 | Severe Gram-negative bacterial infection |
|  | Pediatric patients[14] | 25 | Severe Gram-negative bacterial infection |
|  | Pediatric patients[15] | 46 | Patients with cancer and concurrent bacterial infection (infection type unknown) |
| Sulbactam | Adult HV[16] | 20 | / |
|  | Adult HV[17] | 11 | / |
|  | Adult Patients[18] | 40 | Perioperative prophylaxis for orthopedic surgery patients |
|  | Pediatric patients[19] | 13 | Children with bronchopneumonia, meningitis, lymphadenitis, arthritis, and pharyngitis (bacteria type unknown) |

**Table S5:** Demographics of virtual subjects generated for PBPK simulation of polymyxin-B

| **Number of Subjects** | **Age Range** | **Body Weight (kg)** | **Female (%)** | **Dose** |
| --- | --- | --- | --- | --- |
| **Virtual Adult Population** | | | | |
| 100 | 18 - 70 years | 48–107 | 50 | LD 2.5 mg/kg + 1.5 mg/kg  LD 2.0 mg/kg +1.25 mg/kg |
| **Virtual Pediatric Population** | | | | |
| 1000 | 2 to <18 years | 11 - 67 | 50 | 1.25 mg/kg |
|  | 0 to <2 years | 4 - 12 | 50 | 2.0 mg/kg |

**Table S6:** Demographics of virtual subjects generated for PBPK simulation of amikacin

| **Number of Subjects** | **Age Range** | **Body Weight (kg)** | **Female (%)** | **Dose** |
| --- | --- | --- | --- | --- |
| **Virtual Adult Population** | | | | |
| 1000 | 18 - 70 years | 48–107 | 50 | 15 mg/kg |
| 1000 | 18 - 70 years | 48–107 | 50 | 30 mg/kg |
| **Virtual Pediatric Population** | | | | |
| 1000 | 0 to <18 years | ≥ 40 | 50 | 7.5 mg/kg |
|  |  | ≥ 40 | 50 | 15 mg/kg |
|  |  | < 40 | 50 | 7.5 mg/kg |
|  |  | ≥ 40 | 50 | 15 mg/kg |

**Table S7:** Demographics of virtual subjects generated for PBPK simulation of sulbactam

| **Number of Subjects** | **Age Range** | **Body Weight (kg)** | **Female (%)** | **Dose** |
| --- | --- | --- | --- | --- |
| **Virtual Adult Population** | | | | |
| 100 | 18 - 70 years | 48–107 | 50 | 3000 mg |
| **Virtual Pediatric Population** | | | | |
| 1000 | 0 to <18 years | ≥ 40 | 50 | 1500 mg |
|  |  | < 40 | 50 | 50 mg/kg |

**Figure S1:** Comparison of polymyxin-B, amikacin and sulbactam concentration-time profiles in the blood, lung, heart and skin for adult and pediatric dosing regimens between observed and physiologically based pharmacokinetic modeling

**
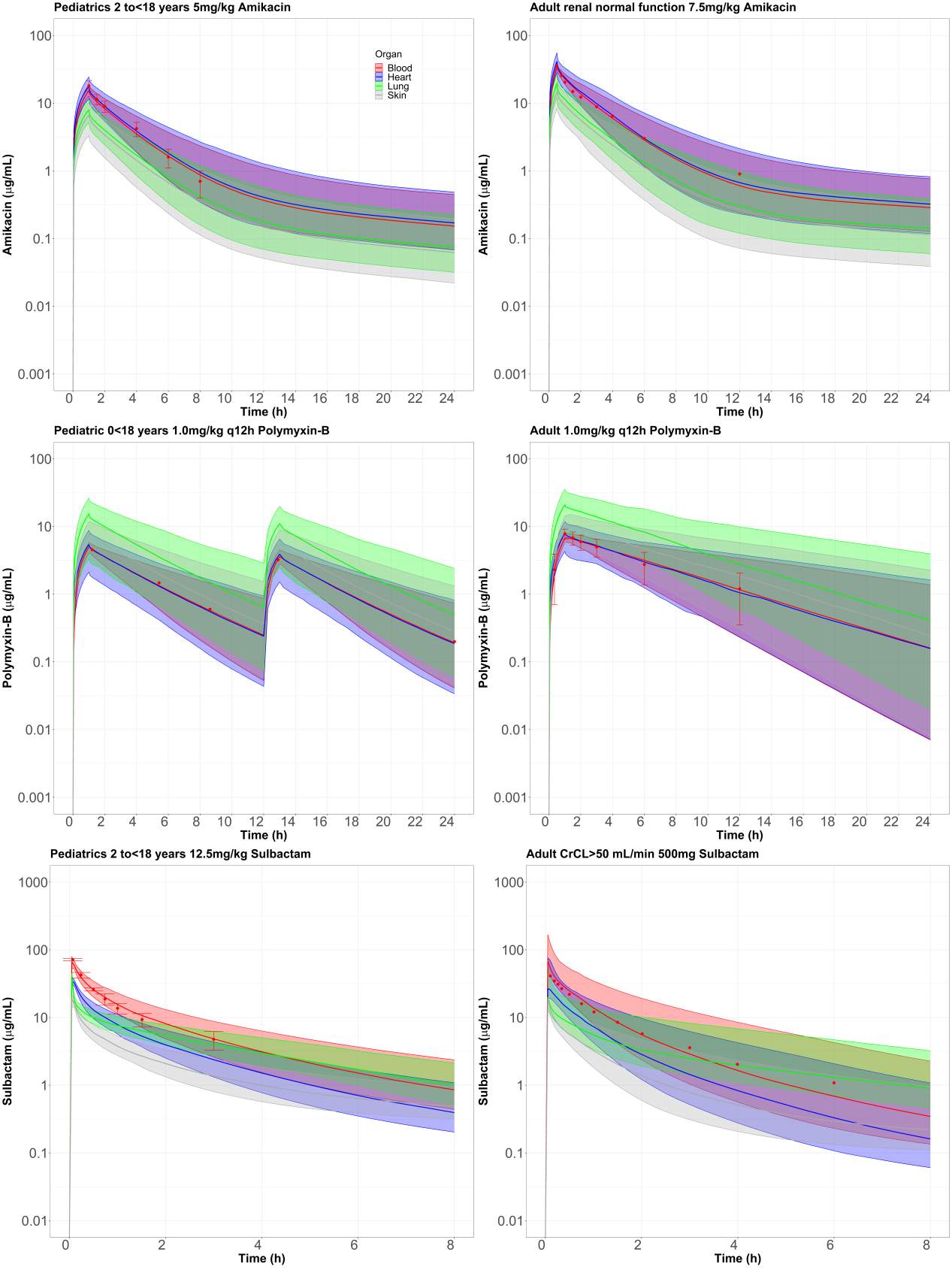
**

**Figure S2:** Comparison of simulations of free polymyxin-B regimens by renal function in adults using population PK and PBPK models


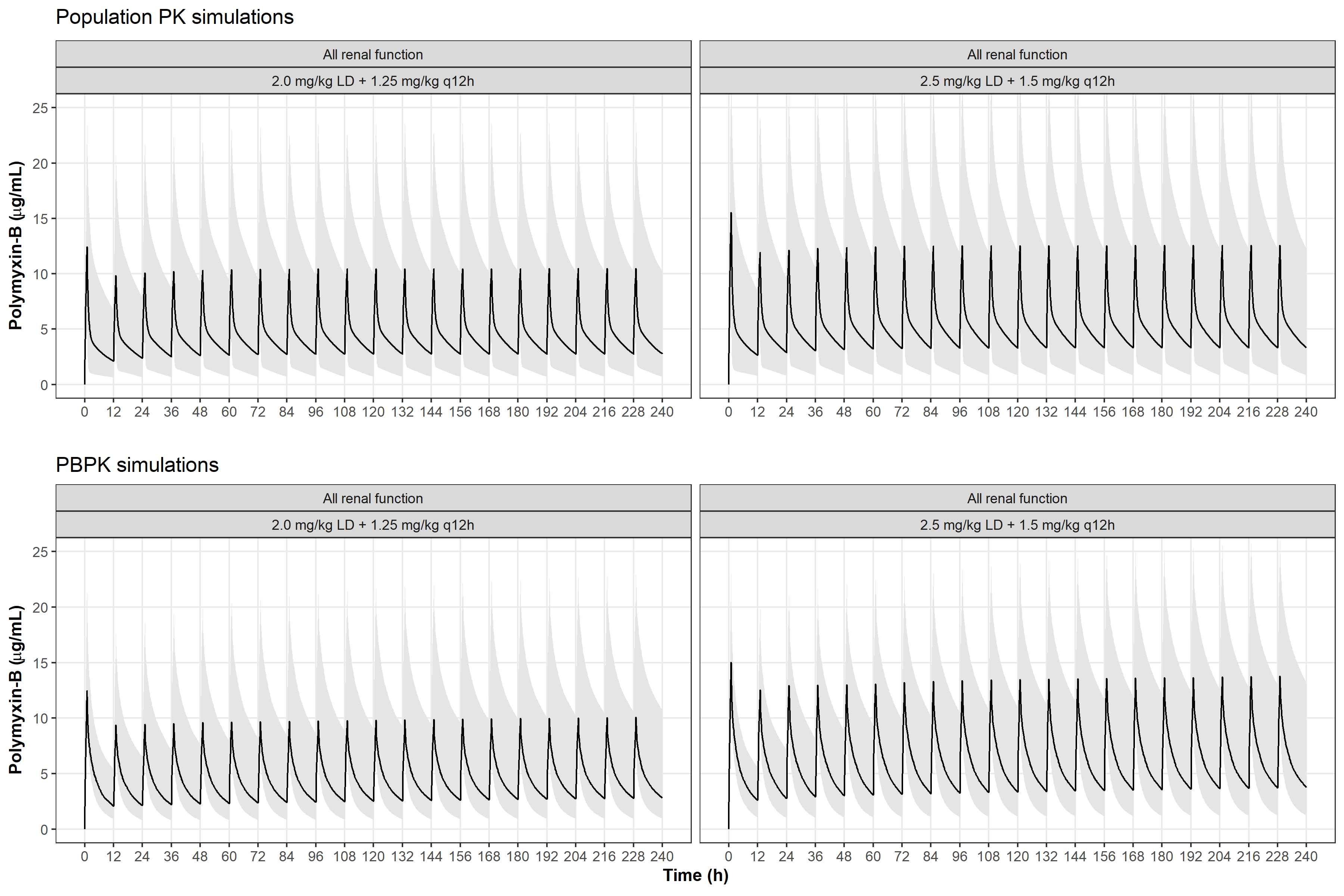


**Figure S3:** Comparison of simulations of total amikacin regimens by renal function in adults using population PK and PBPK models


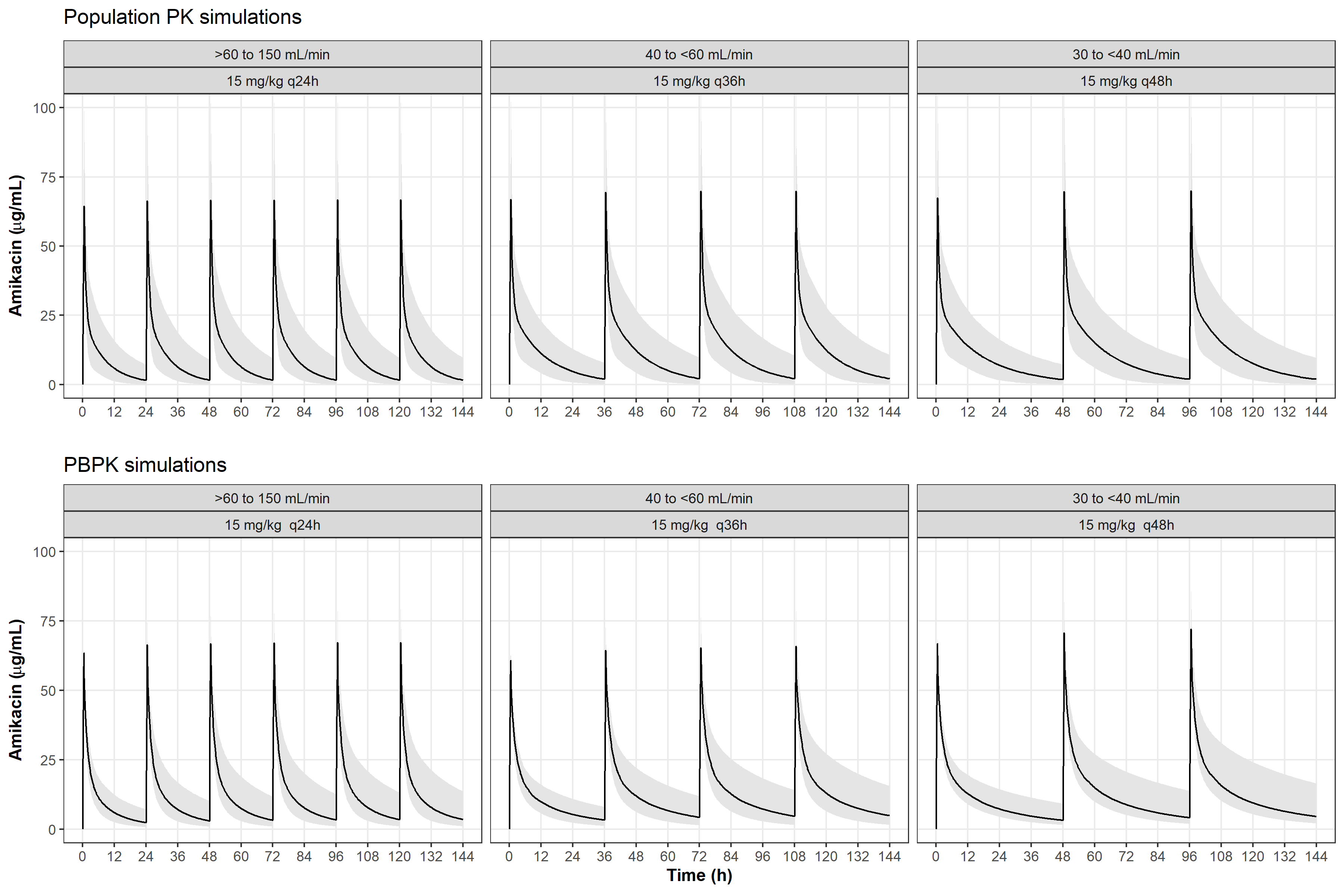


**Figure S4:** Comparison of simulations of free sulbactam regimens by renal function in adults using population PK and PBPK models


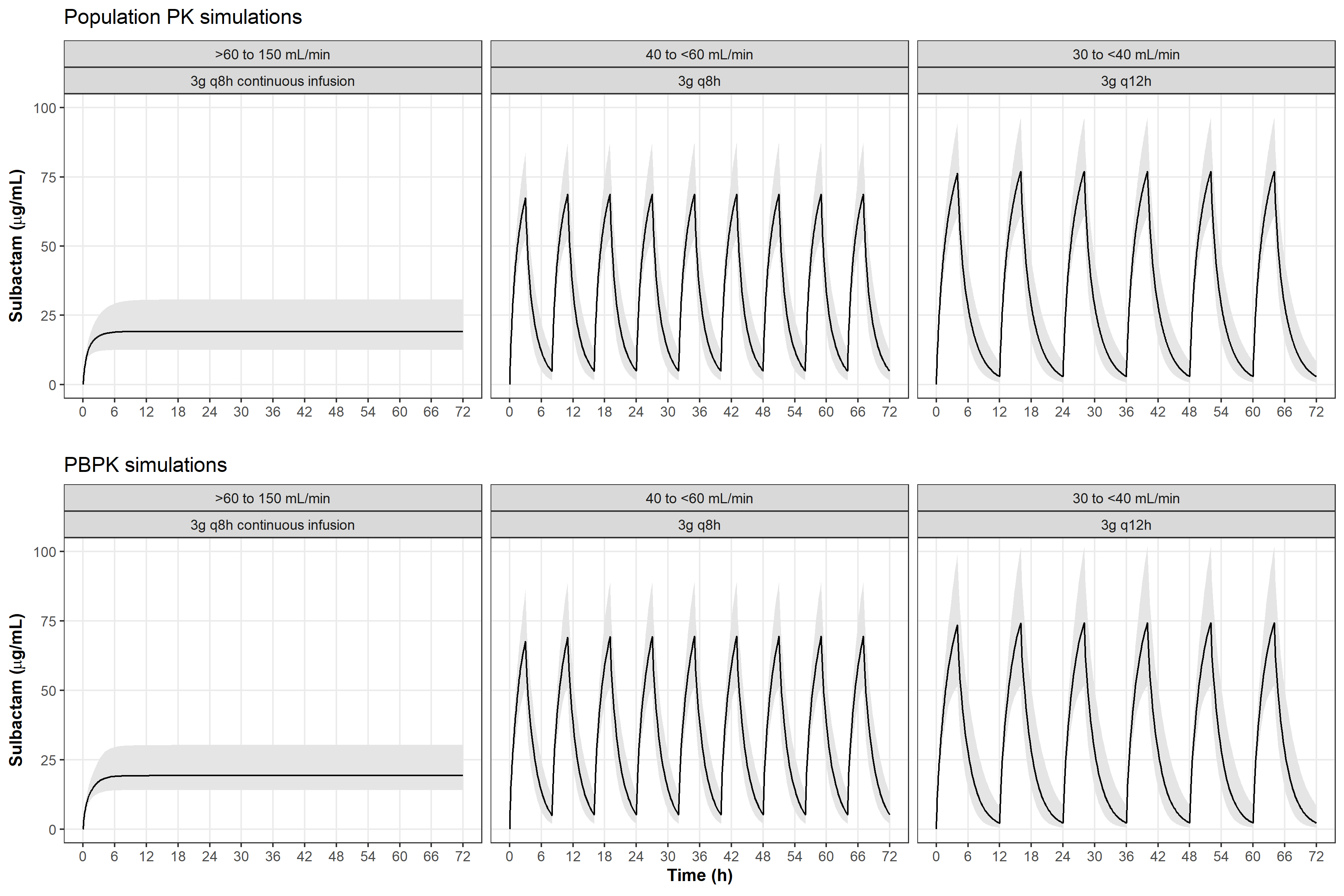


**Figure S5:** Probability of target attainment of amikacin PD index of *f*Cmax/MIC ratio of at least 8 in the blood, lung, heart and skin for dosing regimens in pediatrics and adults by renal function

**
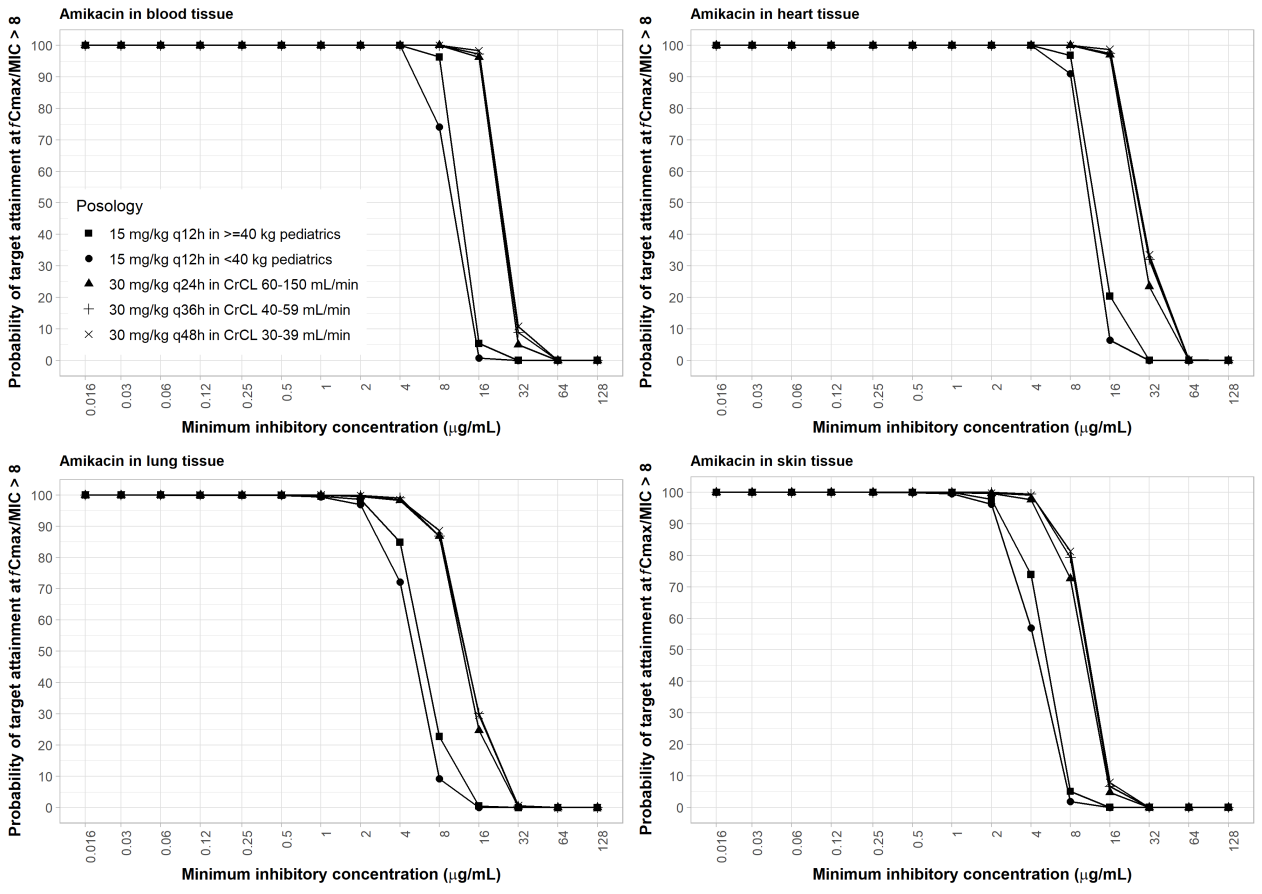
**

**Figure S6:** Probability of target attainment of amikacin PD index of *f*Cmax/MIC ratio of at least 8 in the blood, lung, heart and skin for dosing regimens in pediatrics and adults by renal function

**
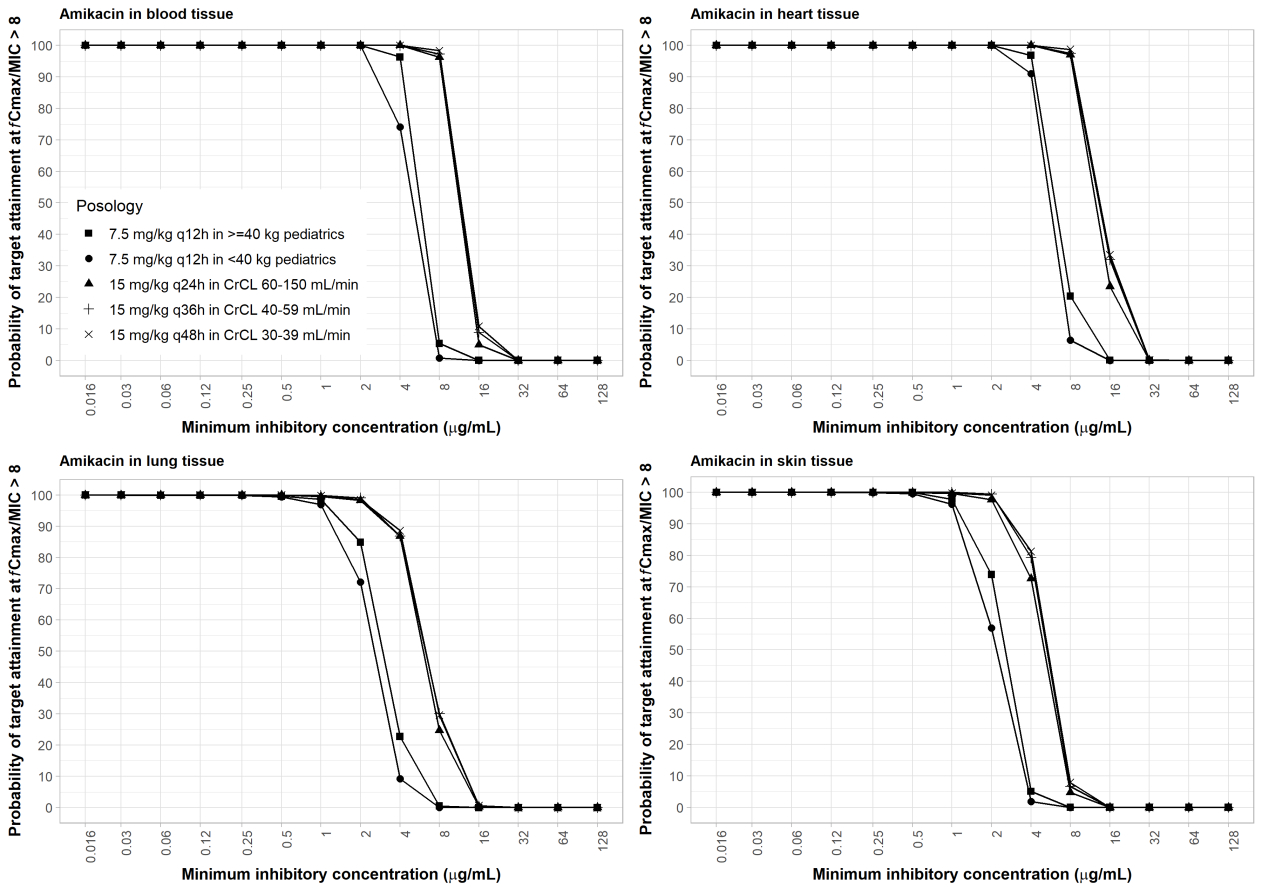
**

**Figure S7:** Probability of target attainment of amikacin PD index of *f*AUC_24_/MIC ratio of at least 80 in the blood, lung, heart and skin for dosing regimens in pediatrics and adults by renal function

**
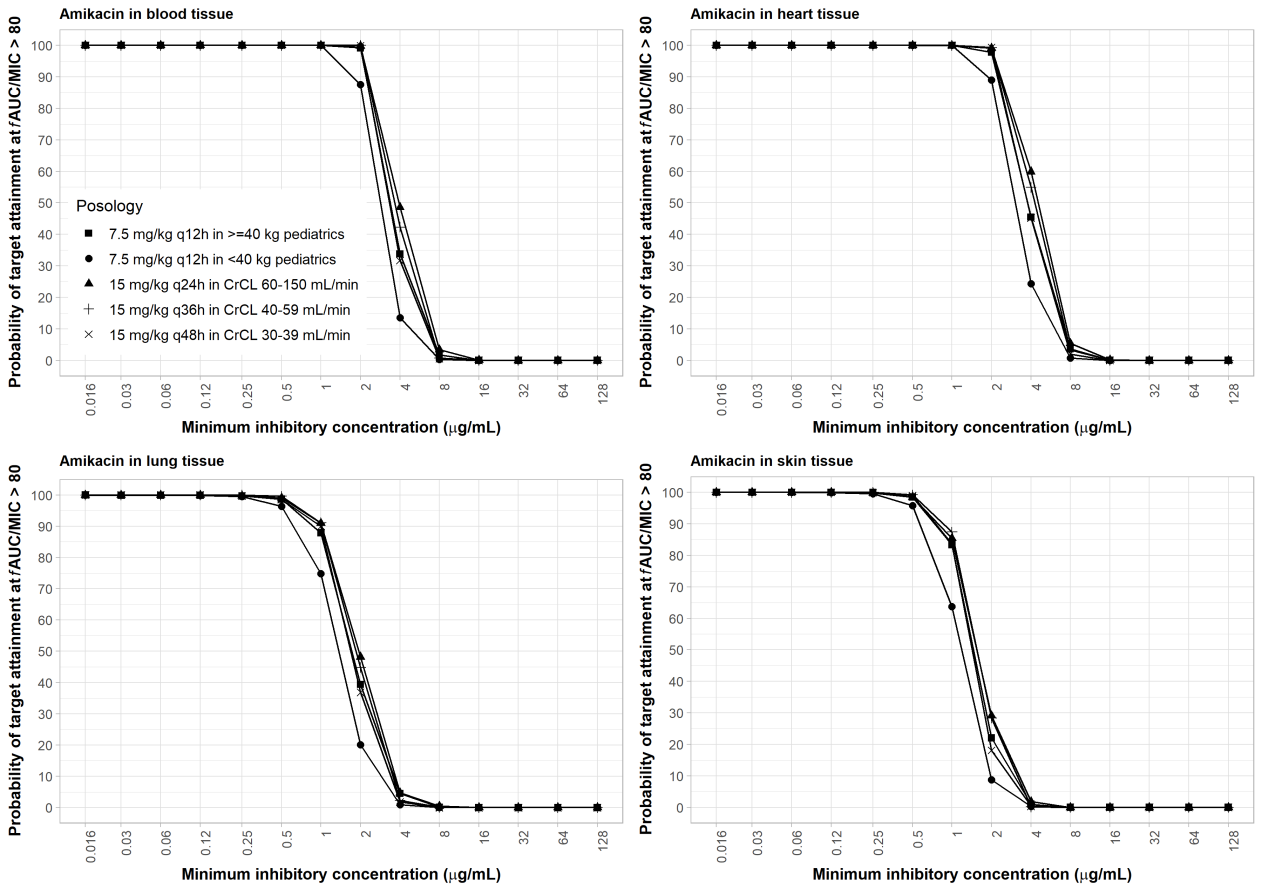
**

**Reference**

1. Zavascki AP, Goldani LZ, Cao G, Superti SV, Lutz L, Barth AL, et al. Pharmacokinetics of intravenous polymyxin B in critically ill patients. Clin Infect Dis. 2008 Nov 15;47(10):1298-304.

2. Cao G, Ali FE, Chiu F, Zavascki AP, Nation RL, Li J. Development and validation of a reversed-phase high-performance liquid chromatography assay for polymyxin B in human plasma. J Antimicrob Chemother. 2008 Nov;62(5):1009-14.

3. Burkin MA, Galvidis IA, Surovoy YA, Plyushchenko IV, Rodin IA, Tsarenko SV. Development of ELISA formats for polymyxin B monitoring in serum of critically ill patients. J Pharm Biomed Anal. 2021 Sep 10;204:114275.

4. Liu X, Yu Z, Wang Y, Wu H, Bian X, Li X, et al. Therapeutic drug monitoring of polymyxin B by LC-MS/MS in plasma and urine. Bioanalysis. 2020 Jun;12(12):845-55.

5. Xu C, Liu X, Cui Y, Huang X, Wang Y, Fan Y, et al. Case Report: Therapeutic Drug Monitoring of Polymyxin B During Continuous Renal Replacement Therapy in Two Pediatric Patients: Do Not Underestimate Extracorporeal Clearance. Front Pharmacol. 2022;13:822981.

6. Garraffo R, Drugeon HB, Dellamonica P, Bernard E, Lapalus P. Determination of optimal dosage regimen for amikacin in healthy volunteers by study of pharmacokinetics and bactericidal activity. Antimicrob Agents Chemother. 1990 Apr;34(4):614-21.

7. Clarke JT, Libke RD, Regamey C, Kirby WM. Comparative pharmacokinetics of amikacin and kanamycin. Clin Pharmacol Ther. 1974 Jun;15(6):610-6.

8. Mahmoudi L, Mohammadpour AH, Ahmadi A, Niknam R, Mojtahedzadeh M. Influence of sepsis on higher daily dose of amikacin pharmacokinetics in critically ill patients. Eur Rev Med Pharmacol Sci. 2013 Feb;17(3):285-91.

9. Taccone FS, Laterre PF, Spapen H, Dugernier T, Delattre I, Layeux B, et al. Revisiting the loading dose of amikacin for patients with severe sepsis and septic shock. Crit Care. 2010;14(2):1-10.

10. Bauer LA, Blouin RA, Griffen WO, Jr., Record KE, Bell RM. Amikacin pharmacokinetics in morbidly obese patients. American journal of hospital pharmacy. 1980 Apr;37(4):519-22.

11. Segal JL, Brunnemann SR, Eltorai IM. Pharmacokinetics of amikacin in serum and in tissue contiguous with pressure sores in humans with spinal cord injury. Antimicrob Agents Chemother. 1990 Jul;34(7):1422-8.

12. Lanao JM, Dominguez-Gil AA, Dominguez-Gil A, Malaga S, Crespo M, Santos F. Pharmacokinetics of amikacin in children with normal and impaired renal function. Kidney Int. 1981 Jul;20(1):115-21.

13. Kafetzis DA, Sianidou L, Vlachos E, Davros J, Bairamis T, Papandreou Y, et al. Clinical and pharmacokinetic study of a single daily dose of amikacin in paediatric patients with severe gram-negative infections. J Antimicrob Chemother. 1991 May;27 Suppl C:105-12.

14. Trujillo H, Robledo J, Robledo C, Espinal D, Garces G, Mejia J, et al. Single daily dose amikacin in paediatric patients with severe gram-negative infections. J Antimicrob Chemother. 1991 May;27 Suppl C:141-7.

15. Cleary TG, Pickering LK, Kramer WG, Culbert S, Frankel LS, Kohl S. Amikacin pharmacokinetics in pediatric patients with malignancy. Antimicrob Agents Chemother. 1979 Dec;16(6):829-32.

16. Ripa S, Ferrante L, Prenna M. Pharmacokinetics of sulbactam/ampicillin in humans after intravenous and intramuscular injection. Chemotherapy. 1990;36(3):185-92.

17. Foulds G, Stankewich JP, Marshall DC, O'Brien MM, Hayes SL, Weidler DJ, et al. Pharmacokinetics of sulbactam in humans. Antimicrob Agents Chemother. 1983 May;23(5):692-9.

18. Wildfeuer A, Mallwitz J, Gotthardt H, Hille E, Gruber H, Dahmen G, et al. Pharmacokinetics of ampicillin, sulbactam and cefotiam in patients undergoing orthopedic surgery. Infection. 1997 Jul-Aug;25(4):258-62.

19. Schaad UB, Guenin K, Straehl P. Single-dose pharmacokinetics of intravenous sulbactam in pediatric patients. Reviews of infectious diseases. 1986 Nov-Dec;8 Suppl 5:S512-7.
